# Computational Analysis of *Balanites aegyptiaca* Phytochemicals as Inhibitors of Human Pancreatic α‐Amylase

**DOI:** 10.1002/cbdv.202503133

**Published:** 2026-02-12

**Authors:** Surendra Kumar Gautam, Rakesh Kumar Paul, Smita Jain, Iqrar Ahmad, Ammar A. Razzak Mahmood, Harun Patel, Penke Vijaya Babu, Muhammad Wahajuddin, Kaisar Raza

**Affiliations:** ^1^ Department of Pharmacy School of Chemical Sciences and Pharmacy Central University of Rajasthan Ajmer India; ^2^ Department of Pharmacology School of Pharmacy and Technology Management SVKM's NMIMS Deemed‐to‐be University Shirpur India; ^3^ Department of Pharmaceutical Chemistry R. C. Patel Institute of Pharmaceutical Education and Research Shirpur India; ^4^ Department of Pharmaceutical Chemistry Prof. Ravindra Nikam College of Pharmacy, Gondur Dhule India; ^5^ Department of Pharmaceutical Chemistry University of Baghdad Baghdad Iraq; ^6^ Department of Pharmaceutical Sciences Tikvah Pharma Solutions Pvt Ltd, IDA‐Cherlapally Hyderabad India; ^7^ School of Pharmacy and Medical Sciences Institute of Cancer Therapeutics University of Bradford Bradford UK

**Keywords:** *Balanites aegyptiaca*, Diabetes Mellitus, Molecular Docking, Molecular Dynamics, Natural Library, Network Pharmacology

## Abstract

*Balanites aegyptiaca* (BA) is a plant of paramount potential for the management of diabetes mellitus. The study investigates in silico studies of natural library compounds including that from BA with the assistance of network pharmacology. Balanitesin (compound **1**) exhibited the highest docking score and binding free energy (∆*G*) values of −14.406 and −125.47 kcal/mol, respectively, and SN0224203 (compound **9**) exhibited the docking score of −13.019 kcal/mol and binding free energy of −128.41 kcal/mol. These were found to be the most potential α‐amylase inhibitor out of the phytoconstituents of BA, whereas the standard compound exhibited the docking score of −12.500 kcal/mol and ∆*G* value of −81.275 kcal/mol. The network pharmacology results also showed that SN0224203 might act as an α‐amylase inhibitor, it was found to be associated with various genes like GCK, VDCC, PIK3, and mTOR and Type II diabetes mellitus pathway. The MDS results showed that the binding of SN0224203 with α‐amylase was more stable as vivid from 50 to 300 ns simulation. Genes. Our results suggest that the compounds of BA were found to be potent against α‐amylase. The findings are promising and suggest further in‐vitro and in‐vivo validation studies of the potent compounds from BA for better diabetes management.

## Graphical abstract

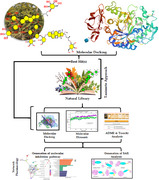

## 1 Introduction

Advancements in cheminformatics have gained particular interest in the drug development process [1]. Methodologies in the in silico drug discovery process utilize several computer algorithms, statistical methodologies, machine learning, and artificial intelligence [2]. As a result, computational biology has emerged as the key paradigm for investigating new possible drug candidates for various diseases [3]. Techniques like structure and ligand‐based drug design play a crucial role in the early identification of drug‐like molecules for several diseases [4]. Discoveries in the field of diabetes have attracted interest in developing new molecules using computational approaches [5, 6]. In the last 10 years, such attempts, especially in diabetes, have resulted in a new synthetic compound, that is, epalrestat (Kinedak), vouching for the promises of the chemoinformatic approach [7].

**FIGURE 1:**
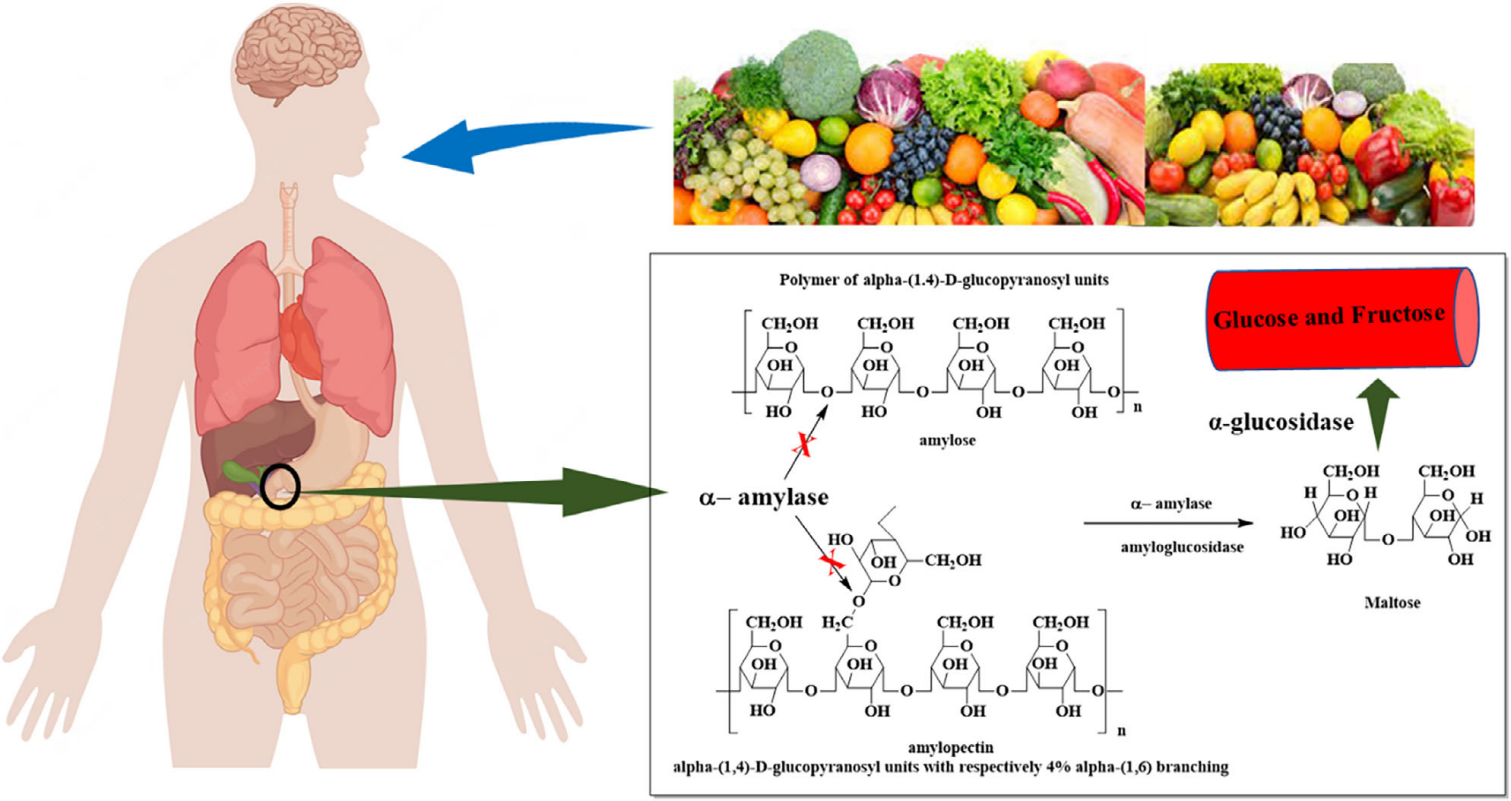
Mechanistic breakdown of carbohydrates via the α‐amylase enzyme. Reproduced from Nadaroglu and Polat [18] with permission from Elsevier.

Type 2 diabetes mellitus (T2DM) is a chronic metabolic disorder characterized by hyperglycaemia resulting from defects in the insulin action, secretion of insulin or both. The rapid increase in the number of people suffering from T2DM is becoming a major health concern for nations worldwide [8]. Diabetes can result in concerning complications of a microvascular and macrovascular nature [9]. According to the World Health Organization (WHO) 2021 report, it is projected that about 422 million people aged 20–79 are prone to T2DM worldwide [10]. There are several known diabetic targets like sodium–glucose cotransporter 2 (SGLT2), α‐amylase, α‐glucosidase, dipeptidyl peptidase‐IV (DPP‐IV), glucagon‐like peptide‐1 (GLP‐1), protein tyrosine phosphatase 1B (PTP 1B) and peroxisome proliferator‐activated receptor (PPAR) [11, 12, 13, 14]. Among these, α‐amylase has become one of the most focused biological targets [15]. These digestive enzymes hydrolyse the dietary carbohydrates to glucose. Thus, inhibiting these enzymes can reduce and control post‐prandial blood glucose levels [16], as shown in Figure 1. The most commonly used inhibitors of α‐amylase are acarbose and miglitol, which are reported to possess side effects such as gastrointestinal disturbance and weight reduction; thus, there is a need for alternative medicine [17].

The WHO also recommends the exploration of traditional medicinal herbs for antidiabetic potential [19]. Plant extracts of more than 1200 species and plant bioactives from more than 400 species have been scientifically explored for T2DM. As per AYUSH guidelines, the plant *Balanites aegyptiaca* (BA) has potential antidiabetic activity [20]. BA belongs to the Zygophyllaceae family and is mainly found in Africa, India (Rajasthan), the Middle East, Nigeria and Asia [21]. Traditionally, BA is reported to be used for the treatment of various diseases such as jaundice, epilepsy, malaria, indigestion, wound healing, haemorrhoids, diarrhoea and asthma. These various pharmacological activities include anti‐inflammatory, antimalarial, anticancer, larvicidal, hepatoprotective, antidiabetic and molluscicidal [22, 23]. The identified phytoconstituents of the BA include balanitin 1–7, saponins, terpenoids, steroids, flavonoids and carbohydrates. According to traditional Egyptian medicine, the leaves, fruits, stems, and fruit mesocarp of BA have been prescribed for the treatment of diabetes [21, 24].

In computational studies, several algorithms like cosine, Tanimoto, and Dice coefficients are used for the similarity search [25]. The cosine and Tanimoto coefficients are based on the angular metric, whereas the Dice coefficient is based on the exhaustive list of distance and similarity measurements [26]. Identification of biologically active compounds using the Tanimoto approach could be an alternative method as it assumes structurally similar compounds should exhibit similar physical chemical properties and yield similar biological activity [27]. The similarity searching are based on the Tanimoto coefficient, which filters large molecules from the database and screens out the fingerprint molecules [28, 29]. In network pharmacology, an in silico approach is more effective for establishing a ‘protein‐targeted compound and disease‐gene’ network for drug discovery and development [30]. It reveals the mechanisms underlying the synergistic therapeutic actions of traditional medicines and the regulation of principles of small molecules in a high‐throughput manner [31]. This advancement of network pharmacology has shifted the paradigm from a ‘one‐target, one‐drug, one disease’ mode to a multiple‐component‐therapeutics/network‐target mode [32].

BA is a native plant of the Central University of Rajasthan; henceforth, the same was selected to generate scientific evidence for the phytoconstituents of the indigenous flora. To investigate the inhibitory potential medicinal compounds found in the plant and natural library on the human pancreatic α‐amylase (HPA) activity using the fingerprint screening of natural libraries compounds, molecular docking, in silico pharmacokinetics (absorption, distribution, metabolism, excretion [ADME]/toxicity), physiochemical properties, molecular dynamics (MD) simulation, and *Homo sapiens* therapeutic target interaction of these network compounds were also determined. Similar in silico exploration of BA phytoconstituents in reference to the supernatural library using Tanimoto approach, molecular docking, MD, in silico pharmacokinetics, and network pharmacology has not yet been reported and produced a scope for future research in these dimensions.

## 2 Results and Discussion

The in silico studies were performed for BA compounds and the phyto‐libraries against α‐amylase activity (PDB ID: 3BAJ, resolution 2.10 Å, *R* value 0.225; accessed on July 29, 2020, version 2.1). The best‐retrieved hits were studied for molecular docking, dynamic simulation, ADME/toxicity studies and network pharmacology.

### 2.1 Selection of Protein Data Bank (PDB)

α‐Amylase is responsible for cleaving α‐1,4 glucan linkages in starch, which are common in plants, animals, and bacteria [33]. Several protein databases are available in the PDB for α‐amylase. The protein structure 3BAJ in the PDB database corresponds to a functional HPA, comprising a single polypeptide chain with 496 amino acids. The enzyme comprises three structural domains: Domain A (covering residues 1–99 and 169–404), Domain B (encompassing residues 100–168) and Domain C (comprising residues 405–496). In Domain A, three active residues such as Asp197, Asp300 and Glu233 are surrounded by an eight‐stranded parallel α/β segment and a nearby chloride ion. Domain B, comprises of residues 100–168, creates a pocket encasing the calcium ion within the α/β‐barrel of Domain A and Domain C (residues 405–496), and folds into an antiparallel β‐barrel of Domain A [33, 34]. The HPA membrane includes glycosidases with two carboxylic acids as the nucleophile and catalyses reaction of acid/base hydrolytic, which proceed via an oxocarbenium ion‐like transition state. The carboxylic acid group of residues Asp197 functions as the catalytic nucleophile, displacing the substrate of aglycon part during the formation of the intermediate covalent. The carboxylic acid residue of Glu233 serves as an acid/base catalyst, thereby contributing to the stabilization of two proposed transition states during hydrolysis. The catalytic residue Asp300 is pivotal for stabilizing the conformation of bound substrates. Activation of HPA occurs allosterically when chloride ions are present. The binding of chloride has an impact on the optimal pH and consequently the activity of these enzymes. The environment within the intestine where HPA operates is rich in chloride ions. Gastric juice and pancreatic excretions are significant sources of fluids containing high levels of chloride ions [35, 36].

### 2.2 Docking Protocol Validation

**FIGURE 2:**
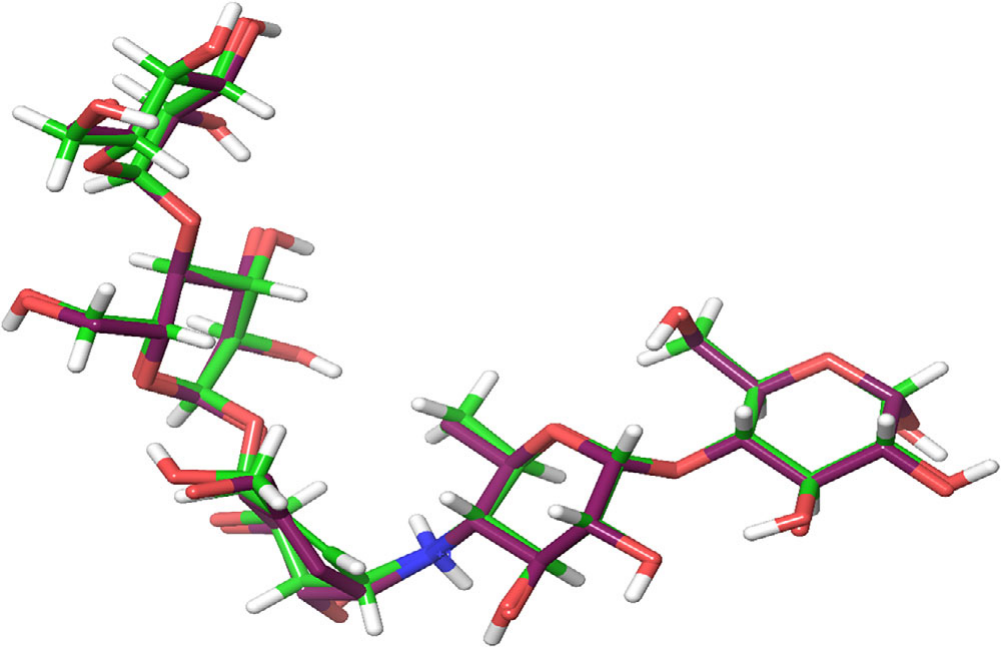
Superimposed structure of acarbose‐derived pentasaccharide.

The molecular docking results were validated through a re‐docking procedure that used the co‐crystallised ligand, an acarbose‐derived pentasaccharide. Using Schrodinger Suite 2023‐1 (Maestro version 13.5), both molecules were superimposed on each other, and the root mean square deviation (RMSD) were obtained. The superimposed structure, that is, acarbose‐derived pentasaccharide, demonstrated the RMSD data of 1.5935 Å and docking score of −16.027 kcal/mol. An RMSD value of less than 2 Å was considered to be in alignment with the conformation [37, 38]. The docked pose of the co‐crystal ligand (green colour) was superimposed with the structure of the acarbose‐derived pentasaccharide ligand (maroon colour), as shown in Figure 2 and other superimpositions of ligands and co‐crystal ligand structures of 1XD0 and 2QV4 listed in Figure S12.

### 2.3 Molecular Docking of BA Compounds and Natural Libraries

**FIGURE 3:**
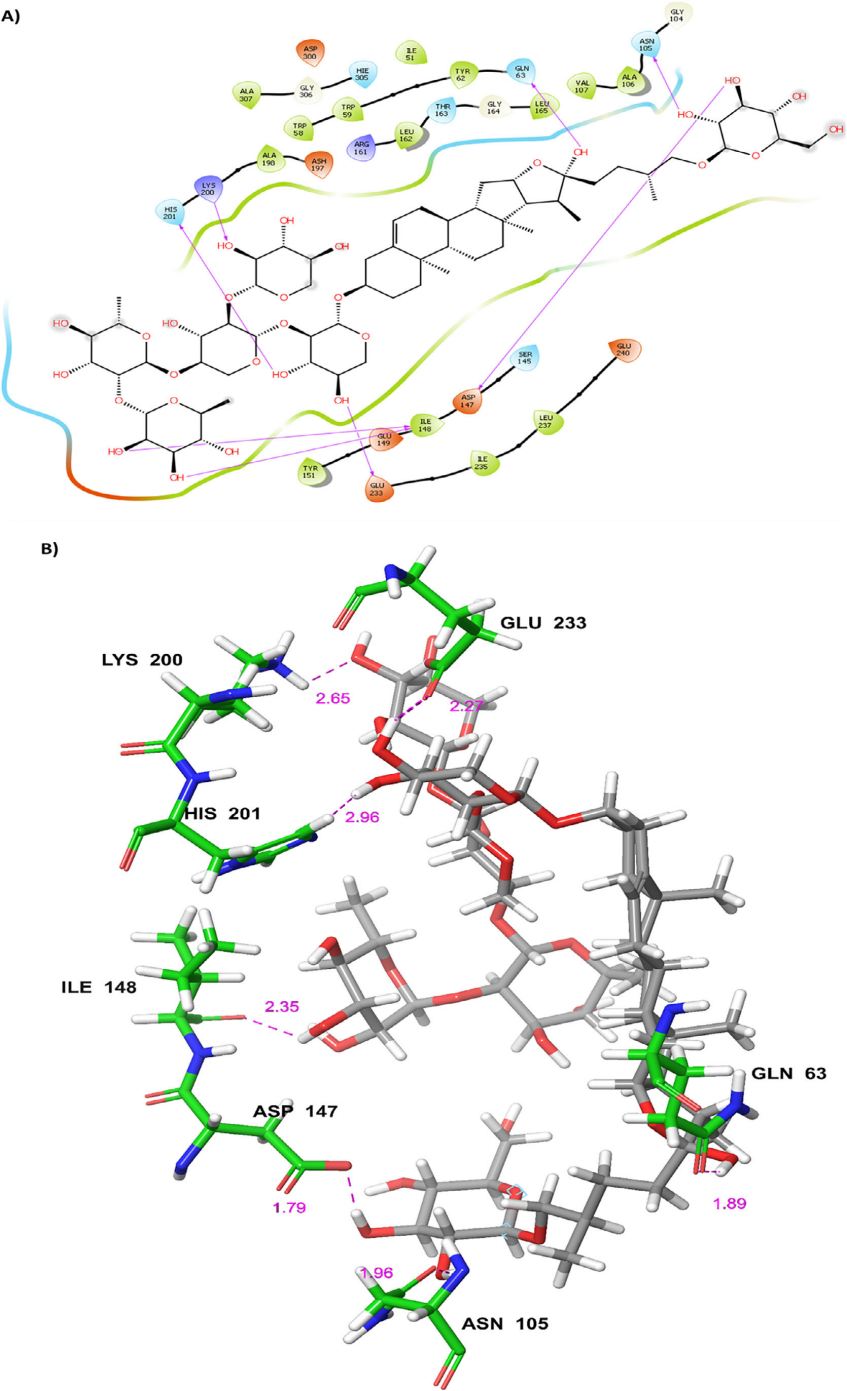
(A) 2D and (B) 3D P‐L contact complexes of Balanitesin (compound **1**) PDB ID: 3BAJ protein (green colour represents amino acids of the grey coloured compound).

The reported compounds of BA were employed for docking‐based approaches using extra precision (XP) mode. The compounds of BA were docked with HPA protein (3BAJ) to identify the binding free energy and dock score. The top five compounds identified from BA were compounds **1**–**5**. Compound **1** showed a good binding free energy (∆*G*) of −125.47 kcal mol^−1^ and docking score of −14.406 kcal mol^−1^ and interacted with residues of Gln63, Asp147, Asn105, Ile148, Ile148, Glu233 Lys200 and His201 by forming hydrogen bonds as shown in Figure 3. Whereas compounds **2**–**5** showed the docking score in the range of −10.929 to −11.797 kcal mol^−1^ and binding energies in the range of −100.808 to −166.261 kcal mol^−1^. Compound **2** interacted with amino acid residues of Glu240, Hie305, Asn152, Gln63, Asp300, Lys200 and Asp356 by forming hydrogen bonding. Compound **3** showed interactions with amino acid residues of Ala50, Glu233, Lys200, Ash197, Arg195, Asp300 and Lys200, Tyr151, Ser108, Tyr52 by forming hydrogen bonding. Compound **4** showed the interaction with amino acid residues like Thr163, Trp59, Gln63, Hie305, Arg197, Glu233, Lys200 and Arg195 by forming hydrogen bonding, whereas compound **5** showed the hydrogen bond interactions with amino acid residues such as Tyr151, Ile148, Arg161, Asp147, Gly164, Asp300, Glu233, Arg197, Hie101 and Lys200. The 2D and 3D protein–ligand complexes for the compounds **2**–**5** have been depicted in Figures S1–S4. The molecular docking of compound **1**, compound **9**, and standard compounds was validated through cross‐docking trials, as well as crystallised ligands from other PDB entries, such as 2QV4 and 1XD0. The docking scores and binding free energies for all PDB entries are listed in Table S5. The correlation plot of ligand using known affinity of MM‐GBSA and *K*_d_/*K*_i_ values of phytocompounds, as shown in Figure S11.

### 2.4 Screening of Natural Compounds Using the Tanimoto Approach

**FIGURE 4:**
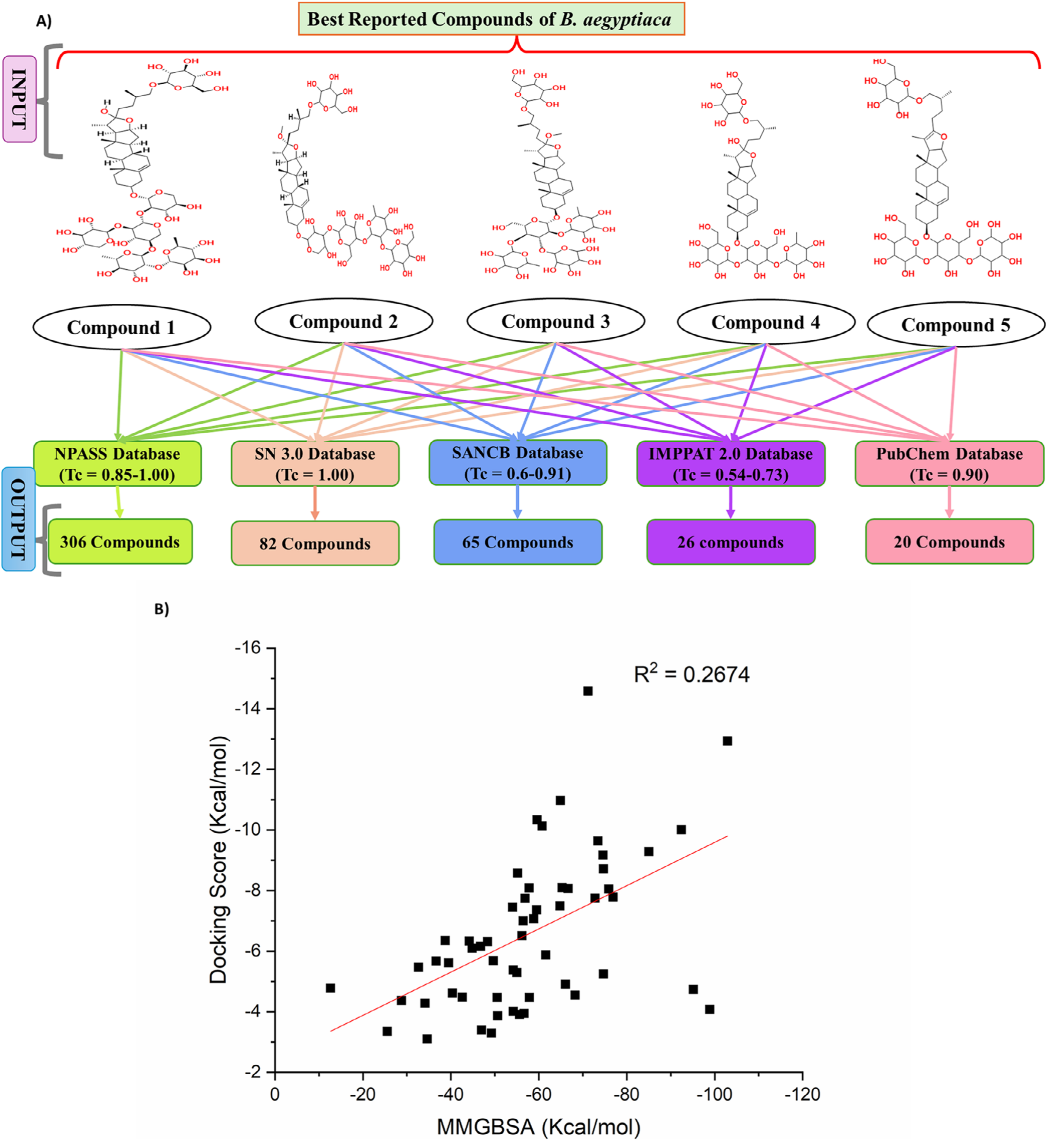
(A) Workflow screening of phyto‐compounds from natural libraries using the similarity (Tanimoto) approach. (B) Known non‐proteinic α‐amylase inhibitor correlation plot of docking score versus MM‐GBSA.

Similar compounds from different natural libraries were screened based on the top‐hit compounds of BA using the similarity (Tanimoto) approach. All the retrieved compounds with *T*_c_ values ranging from the highest (> 1) to the lowest (0.5) were selected as the output for further studies. Based on the similarity approach, a total of 499 compounds were retrieved from various natural library databases, including 20 compounds from PubChem, 26 compounds from IMPPAT, 65 compounds from SANCDB, 82 compounds from SN 3.0, and 306 compounds from NPASS, as shown in Figure 4A. The non‐proteinic α‐amylase inhibitors performed a correlation plot with IC_50_ values of known compounds and docking score, as shown in Figure 4B. The docking score and binding free energy (MM‐GBSA) were represented in Table S6 [39, 40, 41, 42, 43, 44, 45, 46, 47, 48, 49, 50, 51, 52, 53, 54, 55, 56, 57, 58].

**FIGURE 5:**
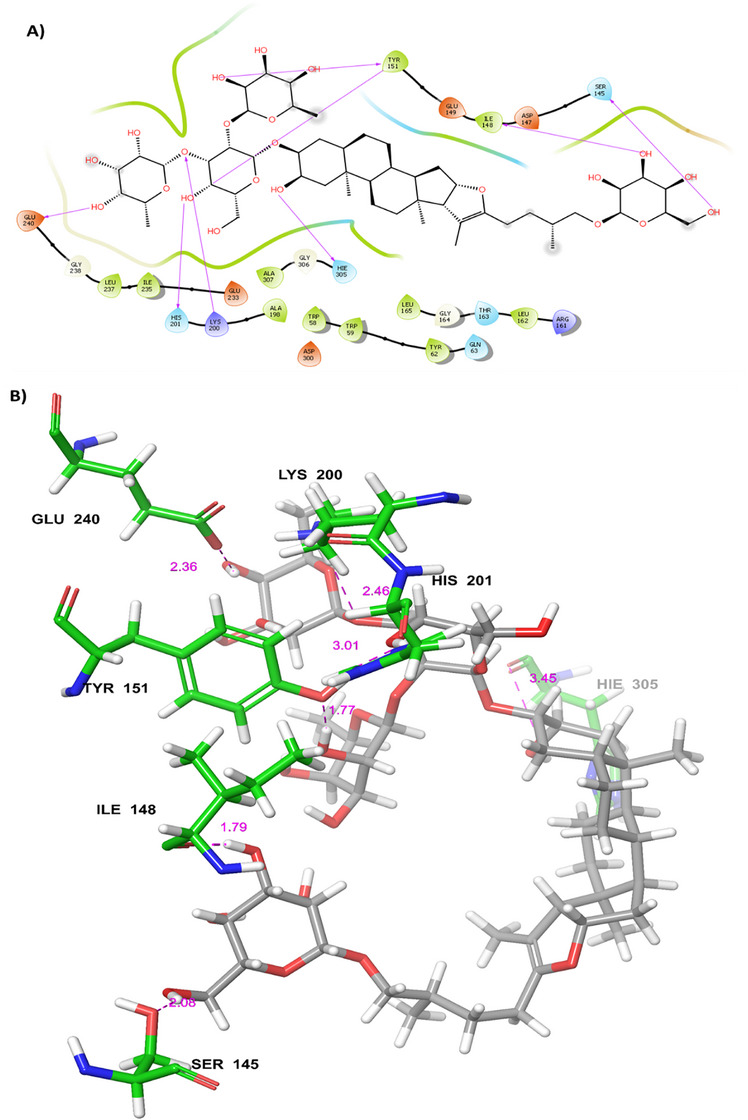
(A) 2D and (B) 3D P‐L complexes of compound 9 (SN0224203) with PDB ID: 3BAJ protein (Green colour represents amino acids of the grey coloured compound).

The XP‐docked hit compounds were studied further for binding energy calculation (MM‐GBSA). The top hit molecules from the natural library were IMPHY001142, PubChem‐10724418, SANC00684, NPC159005, and SN0224203. Compound **9** (SN0224203) showed the best docking score of −13.091 kcal mol^−1^ and ∆*G* of −128.41 kcal mol^−1^ among all the libraries. Compound **9** interacted with amino acid residues such as His201, Glu240, Hie305, Ser145, Ile148, Tyr151 and Lys200 by forming hydrogen bonding, as shown in Figure 5.

**FIGURE 6:**
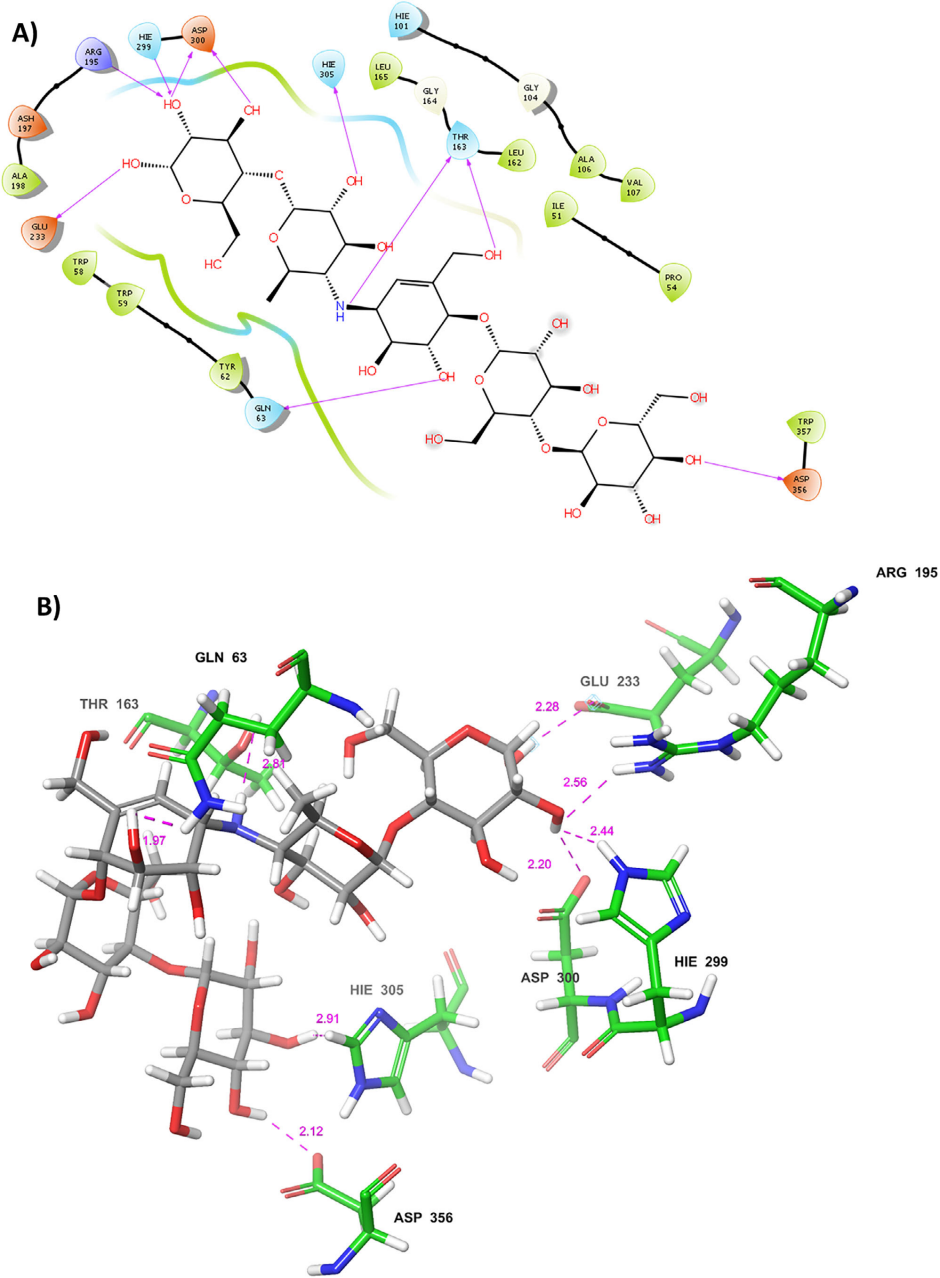
(A) 2D and (B) 3D protein–ligand complexes with acarbose‐derived pentasaccharide (green colour represents amino acids of the grey coloured compound).

**FIGURE 7:**
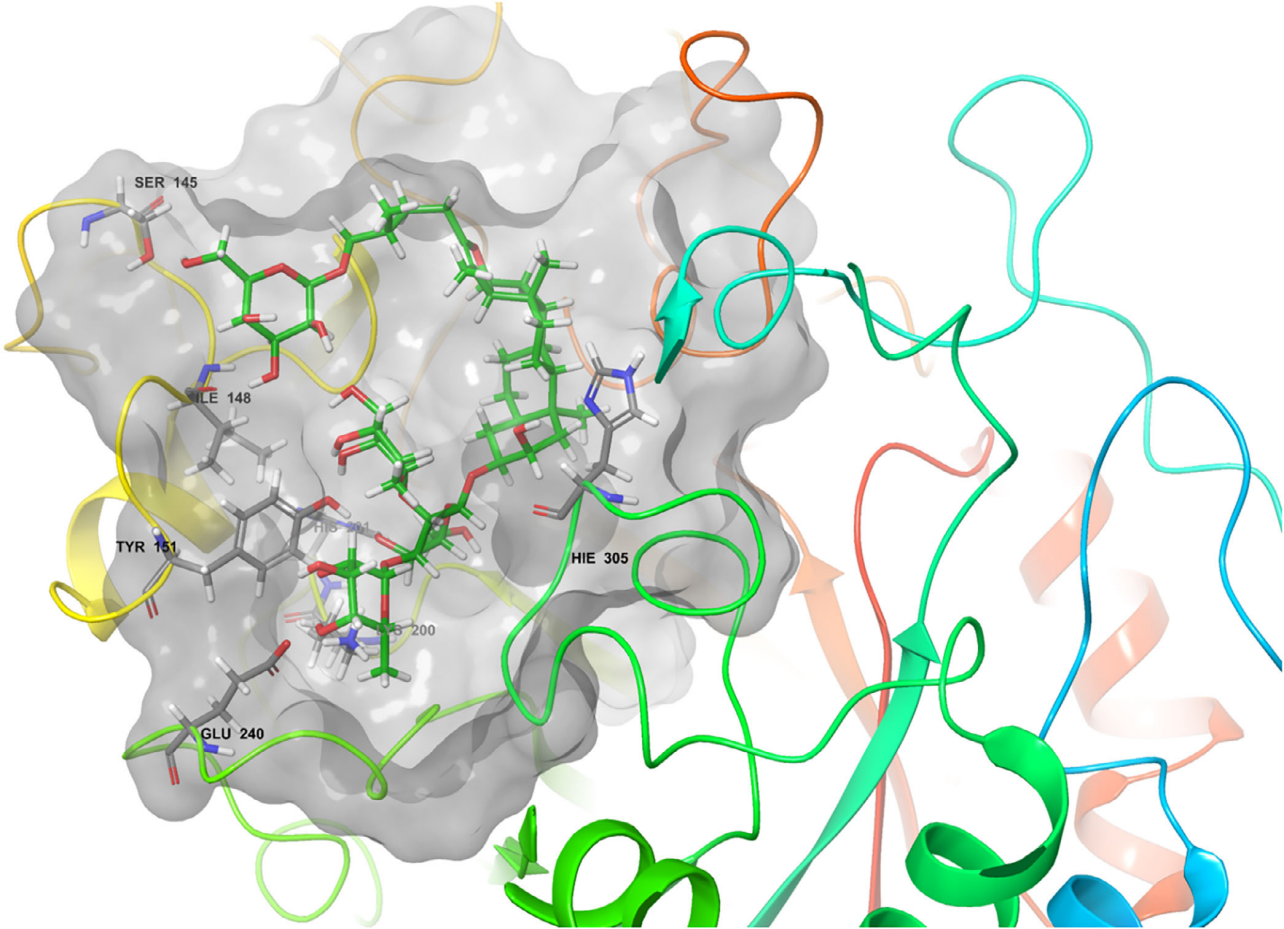
Best hits natural ligands (SN0224203‐green colour and grey colour in amino acid residue) in the catalytic pocket of α‐amylase.

Another compound from natural libraries, compound **6** (IMPHY001142), showed a docking score of −12.864 kcal mol^−1^, ∆*G* of −100.31 kcal mol^−1^ and interacts with amino acid residues of Hie305, Asp300, Glu233, Thr163, Hie299, Arg195, Lys200, Asn105, Ser145, Ash197 by forming hydrogen bonding with 3BAJ protein. Furthermore, the compound from compound **7** (Pub_Chem‐10724418) showed a docking score of −10.730 kcal mol^−1^, ∆*G* of −114.73 kcal mol^−1^ and interacts by forming hydrogen bonding with Thr163, Asp300, Asn105, Tyr151, His201 and Hie299, Arg195, Lys200. Compound **8** (SANC00684) showed DS of −11.372 kcal mol^−1^, ∆*G* of −105.05 kcal mol^−1^ and interacts with the amino acid residues such as Hie305, His201, Asn53, Gln63, Lys200, and Tyr151 by hydrogen bonding and Compound **10** (NPC159005) possesses a docking score of −13.262 kcal mol^−1^ and ∆*G* of −119.29 kcal mol^−1^. Protein–ligand interactions were Hie305, His201, Gln63, Tyr151, Trp59, Thr163, Glu240, Gly239, Tyr62 and Asp356. The 2D and 3D protein–ligand interactions for compounds **6**–**10** have been depicted in Figures S5–S8. The standard compound, acarbose‐derived pentasaccharide, showed the lowest score among all the natural hits with the DS of −12.500 kcal mol^−1^, ∆*G* of −81.275 kcal mol^−1^ and interacts with His201, Glu233, Hie305, Hie305, Thr163, Arg195, Gln63, Asp300, Asp300, Glu233, Hie299, Asp356 by forming hydrogen bonds as shown in Figure 6. All the best‐hit compounds were tabulated with dock score, ∆*G*, and protein–ligand interaction in Table 1. All best‐hit compounds (compounds **2**–**5**) with dock scores, binding energy and protein–ligand complexes are shown in Table S1. The best ligand from the BA, the natural library and the standard compound were depicted in the catalytic pocket of the 3BAJ protein in Figure 7.

**TABLE 1:** Dock score, ∆*G* energy and P‐L complexes of the best topmost hit molecules.

Compounds	Chemical Structures	Dock score (kcal mol^−1^)	Binding free energy (∆*G*) (kcal mol^−1^)	P‐L interactions (H‐bonds) with bond distance
Balanitesin (compound **1**)	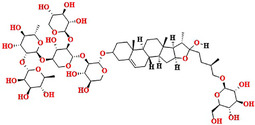	−14.406	−125.47	Gln63 (1.89), Asp147 (1.79), Asn105 (1.96), Ile148 (3.85), Glu233 (2.27), His201 (2.96), Lys200 (2.65)
Compound 9 (SN0224203)	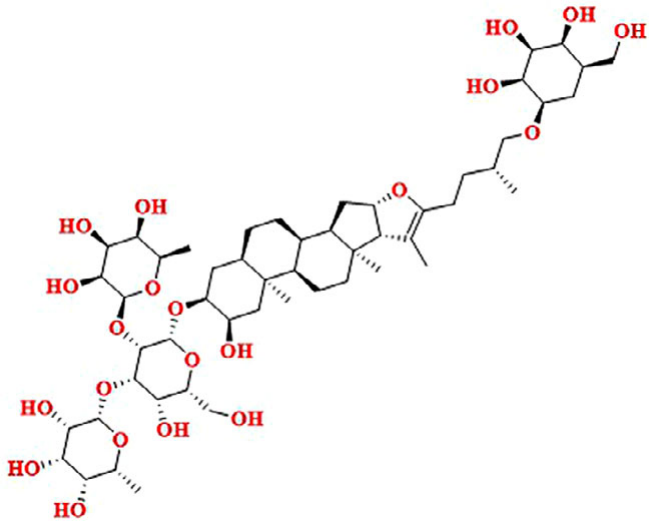	−13.019	−128.41	Ser145 (2.08), Ile148 (1.79), Tyr151 (1.77), His201 (3.01), Lys200 (2.46), Glu240 (2.36), Hie305 (3.45)
Acarbose‐derived pentasaccharide	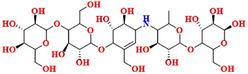	−12.500	−81.275	Glu233 (2.28), Hie305 (2.91), Thr163 (1.97), Gln63 (2.81), Asp300 (2.20), Asp356 (2.12), Arg195 (2.56), Hie299 (2.44)

### 2.5 Analysis of In Silico ADME and Toxicity Studies

In silico pharmacokinetic studies were conducted in a virtual environment to determine the physical and chemical characteristics of the most promising compounds via the Qikprop module. ADME calculates the parameters like central nervous system (CNS), π (carbon and attached hydrogen) component of the solvent accessible surface area (SASA) (PISA), blood–brain barrier (BBB) (log BB), aqueous solubility (log *S*), weakly polar component of the SASA (WPSA), Cl log *S*, apparent Caco‐2 permeability in nm/s (Caco), MDCK, skin permeability (log *K*_p_), human oral absorption (HOA), % HOA, and rule of five. All these parameters were found to fall within the acceptable range. The predicted ADME parameters are presented in Table 2. However, some calculated Qikprop properties of the natural compounds fall outside the range. Parameters like CNS prediction are related to the BBB, and Madin‐Darby canine kidney (MDCK) are related to oral absorption. All the best hits were CNS inactive with negative BBB and lower MDCK values, indicating that the compounds are polar. The descriptors PISA, which is the WPSA and π (carbon and attached hydrogen) component of the SASA, are related to the SASA (range 300–1000). All the hits showed lesser values for WPSA and PISA due to the highly polar nature of the compounds. Another quantitative multiple linear regression‐based descriptor and % HOA were found to be poor for all the natural hit molecules. The aqueous solubility of all the natural hits was found to be within the acceptable range except for the standard compound; hence, both the screened natural compounds were aqueously soluble. Similarly, the conformation‐independent predicted aqueous solubility (Cl log *S*) for all the natural hits were within the range. The Caco permeability for all hit compounds was found to be poor due to the compounds' polar nature. The values of Jorgensen's rule of three and Lipinski's rule of five suggested that all the compounds were in the acceptable range. Among the natural compounds (compounds **1** and **2**), compound **9** were found to lie in the acceptable range for the predicted skin permeability. ProTox‐II is a virtual platform for assessing chemical structure toxicity predictions. The predicted toxicity classes for the best‐hit molecules were 3 and 4, representing the minimum oral toxicity. Amongst the SN00224203 and Balanitesin (compound **1**), SN00224203 was found to be less toxic, and the predicted toxicity with LD_50_ value of 1200 mg/kg, while the LD_50_ of Balanitesin was predicted to be 55 mg/kg. In addition, the acarbose‐derived pentasaccharide (reference) compound belongs to the predicted Class 4. The toxicological properties prediction for various targets, including organ toxicity (hepatotoxicity) was predicted for the top best compounds within the range of 0.93%–0.95%; thus, predicted as nontoxic. The observed toxicity endpoints for carcinogenicity, immunogenicity, mutagenicity, and cytotoxicity were within the accepted accuracy range of 0.63%–0.79%. The ADME/toxicity profiles of the compounds **2**–**10** are shown in Table S2.

**TABLE 2:** In silico ADME and toxicity studies of the best‐hit molecules and the standard compound.

Descriptors	Balanitesin (compound 1)	Compound 9 (SN0224203)	acarbose‐derived pentasaccharide
CNS (−2 inactive/+2active)	−2	−2	−2
PISA (0–450)	24.905	2.436	8.378
WPSA (0–175)	0	0	0
log *S* (−6 to 0.5)	−1.246	−1.252	1.982
Cl log *S* (−6.5 to 0.5)	−4.982	−5.083	1.827
log BB (−3 to 1.2)	−9.548	−4.72	−6.944
PCaco (< 25 poor, > 500 great)	0.027	6.724	0.008
PMDCK (< 25 poor, > 500 great)	0.006	2.225	0.002
log *K*_p_ (−8.0 to −1.0)	−9.085	−5.074	−11.544
HOA (1, 2, 3)	1	1	1
%HOA (> 80 high, < 25 poor)	0	0	0
ROF (max 4)	3	3	3
ROT (max 3)	2	2	2
Predicted toxicity LD_50_ (mg/kg)	55	1200	2000
Predicted toxicity class	3	4	4
Hepatotoxicity (balance accuracy 0.93)	0.95	0.95	0.65
Carcinogenicity (balanced accuracy 0.81)	0.74	0.79	0.84
Immunotoxicity (balanced accuracy 0.75)	0.99	0.99	0.99
Mutagenicity (balanced accuracy 0.84)	0.98	0.95	0.76
Cytotoxicity (balanced accuracy 0.85)	0.71	0.57	0.70

### 2.6 MD Studies

MD simulation was carried out using the Desmond package software for 500 ns. To evaluate the equilibration of the systems, we plotted graphs of potential energy and temperature versus time. These plots allowed us to visually assess the stability and convergence of the systems during the equilibration phase. A stable potential energy plot, showing minimal fluctuations over time, indicates that the system has reached a low‐energy, equilibrated state. Similarly, the temperature versus time plot helps in confirming that the system maintained the target temperature with minimal deviations throughout the simulation. The potential energy and temperature versus time graphs are provided in Figures S9 and S10, respectively. The equilibration data for the complexes demonstrate that the systems have reached a stable and equilibrated state. The average potential energy for the three complexes was found to be −71 170.41 kcal mol^−1^ for the 3BAJ–compound **1** complex, −70 926.53 ± 143.28 kcal mol^−1^ for the 3BAJ–SN0224203 complex, and −70 899.47 ± 133.37 kcal mol^−1^ for the 3BAJ–Std complex. These low potential energy values indicate that each system has settled into a stable configuration. The temperature data further supported this interference. The average temperatures for all three systems were observed to be close to the target value of 298 K, with values of 298.67 ± 1.98, 298.66 ± 1.88, and 298.71 ± 1.89 K for the 3BAJ–compound **1**, 3BAJ–SN0224203, and 3BAJ–Std complexes, respectively. Overall, the stable potential energy, consistent temperature values, and low standard deviations confirmed proper equilibration during the MD simulations.

**FIGURE 8:**
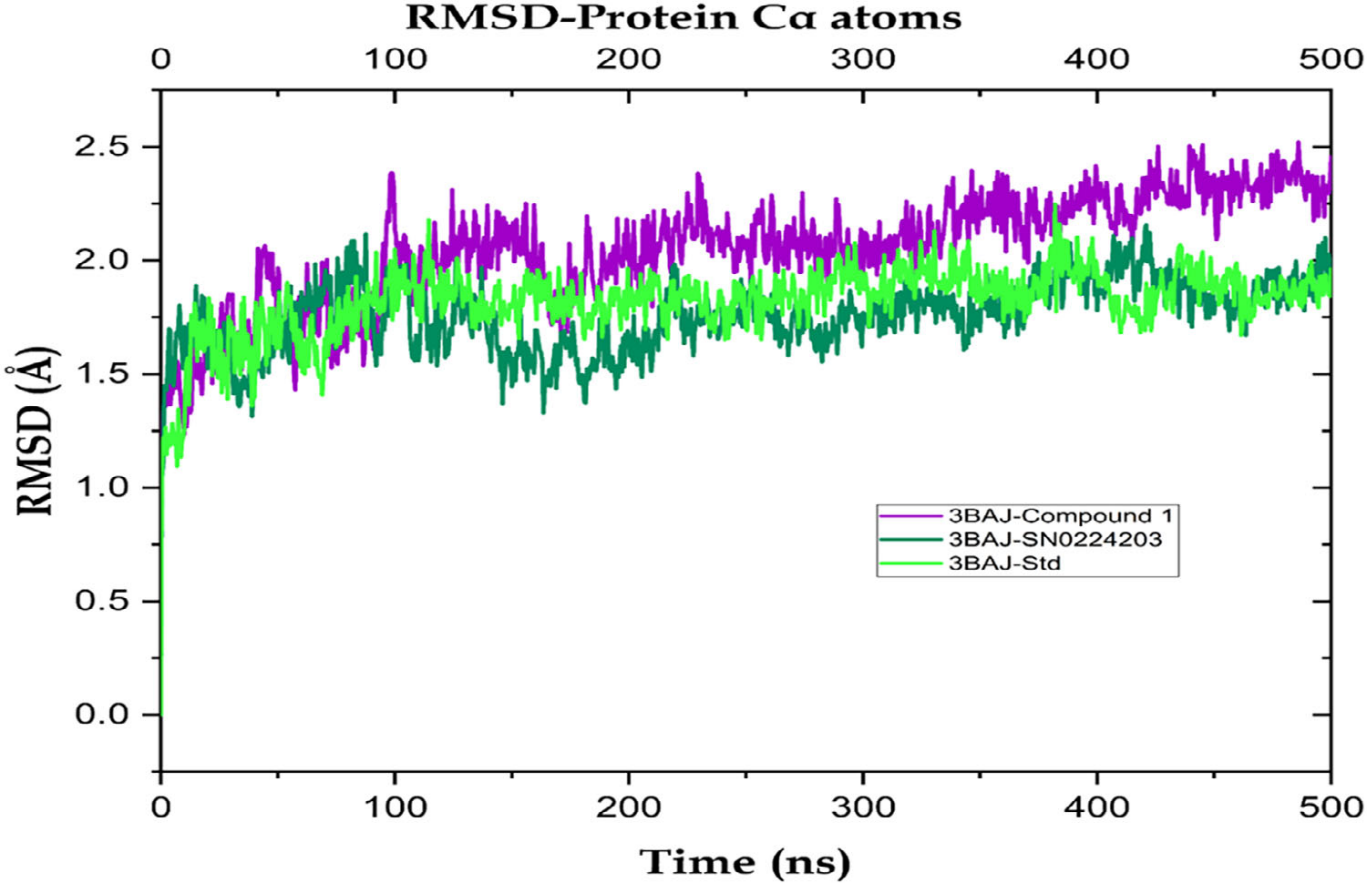
Root mean square deviation (RMSD) of Cα of the protein receptor shown in green colour, standard compound (purple colour) with compound **1**, and RMSD of protein receptor (green colour), standard compound (acarbose‐derived pentasaccharide) and ligand shown in dark green colour with compound **9** (SN0224203).

**FIGURE 9:**
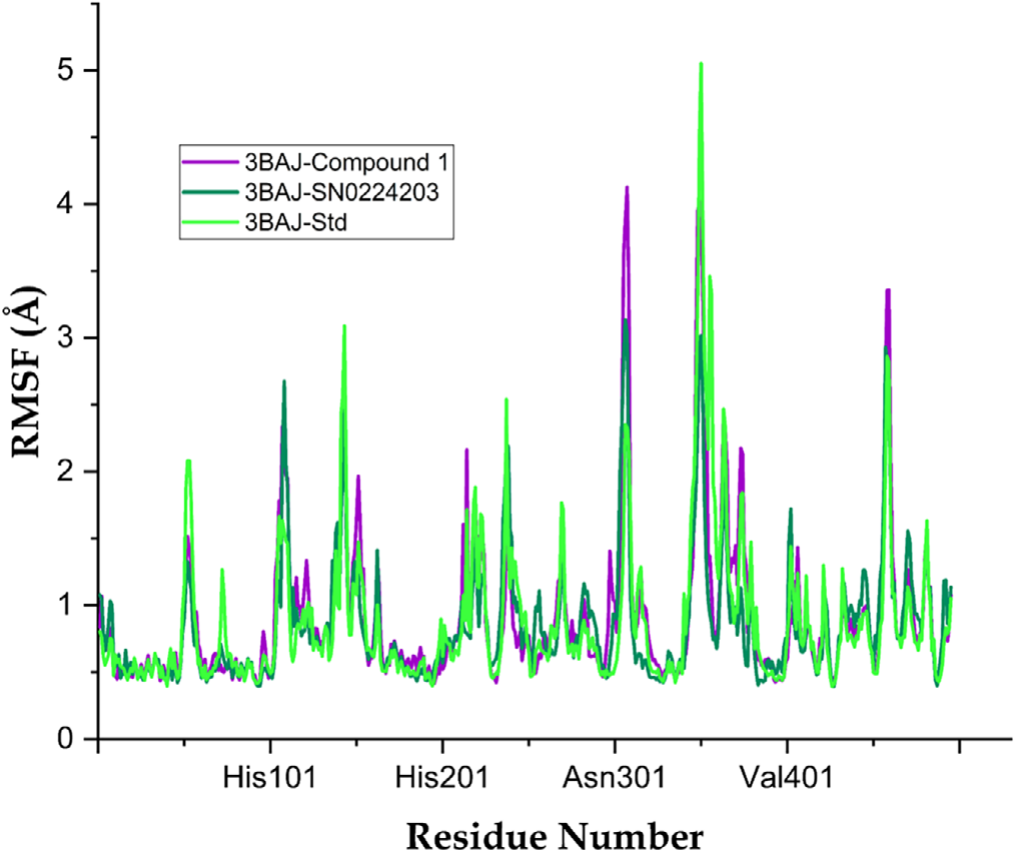
Root mean square fluctuations (RMSF) of Cα of protein receptor (green colour) standard compound and ligand (dark green colour) with compound **9** (SN0224203) and purple colour with compound **1**.

Computational studies, specifically MD simulations, were performed to examine the dynamic properties and binding affinities of compounds **1** and **9** (SN0224203) in comparison to a standard compound, when bound to the target protein. Key parameters assessed comprised RMSD, root mean square fluctuation (RMSF) and hydrogen contact ligand analysis. The protein–ligand complexes atoms were calculated in the system for Balanitesin (compound **1**) as 21 928 atoms, compound **9** (SN0224203) as 21 892 atoms and acarbose‐derived pentasaccharide as 21 876 atoms. The RMSF, RMSD and protein–ligand contacts analysed the structural changes in the complex and dynamic behaviour. The RMSD measures the average change in displacement of 3BAJ Cα atoms between the prepared protein with ligands. The RMSF value is used to characterize local changes along the protein chain after binding with ligands. The average RMSD values for the simulated systems indicated stable binding throughout the trajectory. The 3BAJ–compound **1** complex showed an average RMSD of 1.89 Å with a deviation of ±0.20 Å, while the 3BAJ–SN0224203 complex displayed a slightly lower average of 1.64 Å (±0.18 Å). In comparison, the 3BAJ–standard complex exhibited an average RMSD of 1.95 Å with a deviation of ±0.31 Å. The RMSD plot for the complexes is shown in Figure 8. Similarly, the RMSF profiles supported the overall stability of the complexes. The 3BAJ–compound **1** complex recorded an average RMSF of 0.83 Å (±0.50 Å), whereas the 3BAJ–SN0224203 complex demonstrated a slightly reduced fluctuation with an average of 0.73 Å (±0.53 Å). The 3BAJ–standard complex showed an average RMSF of 0.85 Å accompanied by a deviation of ±0.63 Å. The complexes were found to be more stable at various time intervals with minor fluctuations. In RMSF plots, most regions were below 1.5 Å, as shown in Figure 9. The average and standard deviation values for all complexes are tabulated in Table S7.

**FIGURE 10:**
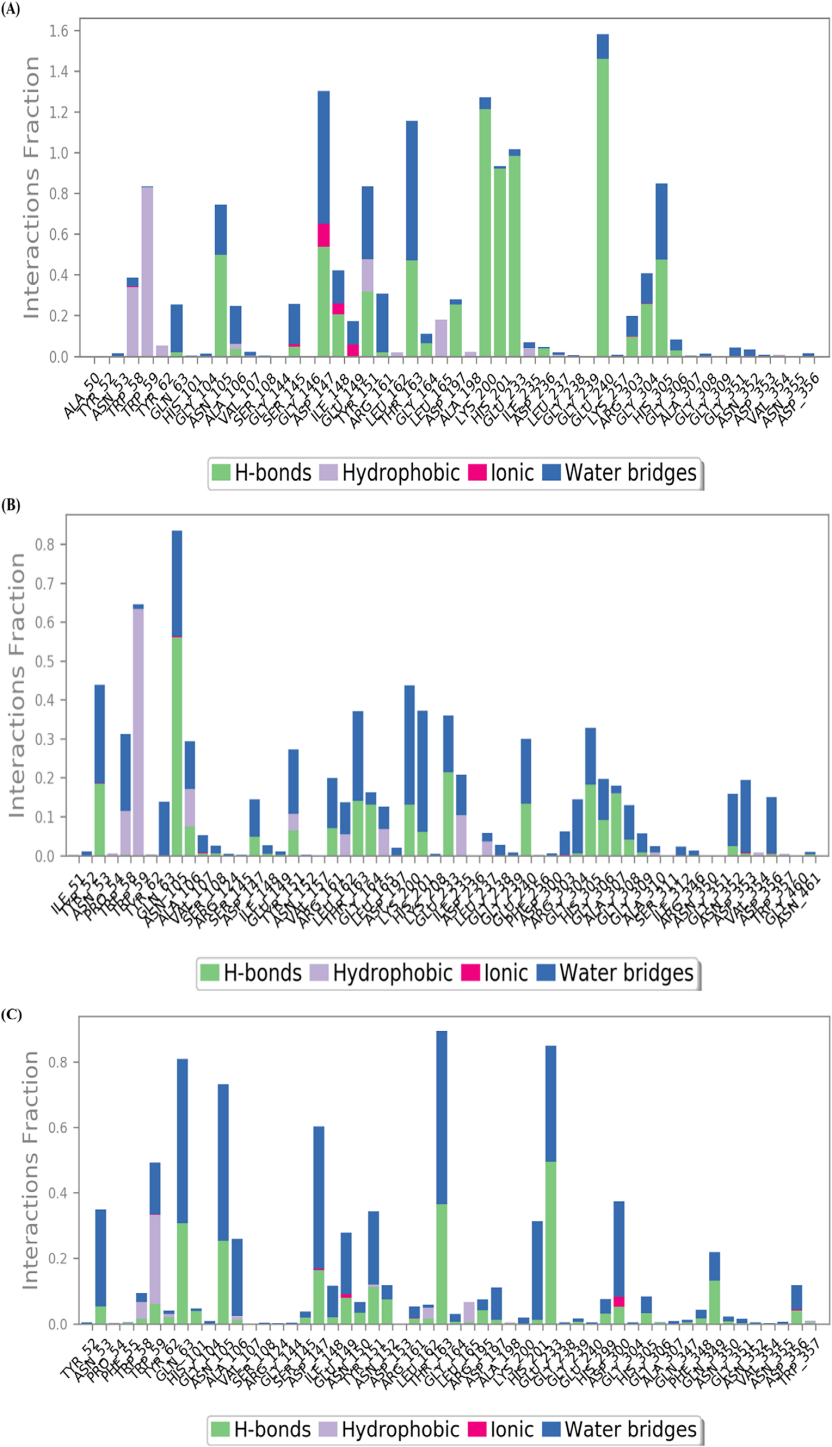
(A) Protein–ligand interaction of acarbose‐derived pentasaccharide with compound **1** complexes; (B) protein–ligand interaction of acarbose‐derived pentasaccharide with compound **9** (SN0224203) complexes; (C) protein–ligand interaction of acarbose derived pentasaccharide with reference compound complexes.

During the MD trajectories simulation of the 3BAJ–compound **1** complex, the hydrogen bonds interactions like Gln63, Asn105, Arg161, Ser145, Asp147, Ile148, Ala106, Ser108, Tyr151, Thr163, Gly164, Asp197, Lys200, His201, Glu233, Asp236, Glu240, Arg303, Gly304, His305, Gly306, Ala307 and water bridge interactions were with amino acid residues Tyr52, Trp58, Gly104, Val107, Ser145, Asp147, Ile148, Leu237, Gly238, Asn105, Ala106, Glu149, Tyr151, Arg161, Thr163, Gly164, His201, Glu233, Asp197, Lys200, Ile235, Glu240, Lys257, Arg303, Gly304, Gly308, Gly351, His305, Gly306, Asn352, Asp353 and Asp356. Furthermore, hydrophobic interactions were with residues Trp58, Ala106, Trp59, Tyr151, Leu162, Tyr62, Ala198, Ile235, Ala307, Val354 and ionic bonds interacted with amino acid residues Ser145, Asp147, Ile148 and Glu149 as shown in Figure 10A. Similarly, for complex 3BAJ–SN0224203, the RMSD was found to be below 2.0 Å, as shown in Figure 8. The complexes of 3BAJ–SN0224203 were found to be stable time intervals with some fluctuations. In the RMSF plot (Figure 9), fluctuations were observed in some regions with RMSF values below 2.5 Å. Several interactions of 3BAJ–SN0224203 complex showed hydrogen bonding with amino acid residues Asn53, Gln63, Asn105, Ala106, Val107, Ser108, Asp147, Tyr151, Arg161, Thr163, Gly164, Lys200, His 201, Glu233, Glu240, Gly304, Gly306, Ala307, Gly308, Gly309, Asn352, His305, Asp353 and hydrophobic interaction with amino acid residues Trp58, Ala106, Tyr151, Leu162, Trp59, Leu165, Ile235, Leu237, Val354, Trp357. Furthermore, water bridge interaction with residues Tyr52, Asn53, Trp58, Gln63, Ala106, Asn105, Val107, Ser108, Arg124, Asp147, Thr163, Asp197, Arg161, Leu162, Gly164, Gly304, Gly306, Leu165, Lys200, His201, Lys208, Glu233, Ile235, Leu237, Gly238, Gly239, Glu240, Asp300, Ile148, Glu149, Arg303, Ala307, Gly308, Gly309, Ala310, Ile312, Arg346, Asn352, Asp356, Asp353, Asn461 and one ionic bond interact with amino acid residue Asn105, Val107, and Asp353 as shown in Figure 10B. Furthermore, the MD for the standard compound complex, the RMSD plot Figure 8, depicts that the protein–ligand RMSD was stable for 400 ns and some fluctuations were observed throughout the simulation of 500 ns. In the RMSF plot (Figure 9), it is evident that most regions are below 1.6 Å, with some observed fluctuations. During the MD trajectories simulation of acarbose‐derived pentasaccharide with 3BAJ protein complex, the hydrogen bonds interactions like Asn53, Phe55, Trp59, Tyr62, Asn105, Gln63, Ile148, Glu149, His101, Ala106, Ser145, Asp147, Asn152, Arg161, Leu162, Thr163, Gly164, His201, Glu233, Asn150, Tyr151, Arg195, Asp197, His299, Asp300, Phe348, Gln349, His305, Gly306, Asp356 and hydrophobic bond interacts with amino acid residues Leu165, Trp59, Tyr62, Ala198, Trp58. Furthermore, water bridge interactions were with amino acid residues Trp58, Trp59, Ala106, Tyr151, Leu162, Leu165 and ionic bond interacts with residues Asp147, Glu149, Arg161, Asp300 and Asp356 as shown in Figure 10C.

### 2.7 Network Pharmacology

#### 2.7.1 Identification of Drug‐Disease‐Targeting and Analysis of Protein–Protein Interaction Network

**FIGURE 11:**
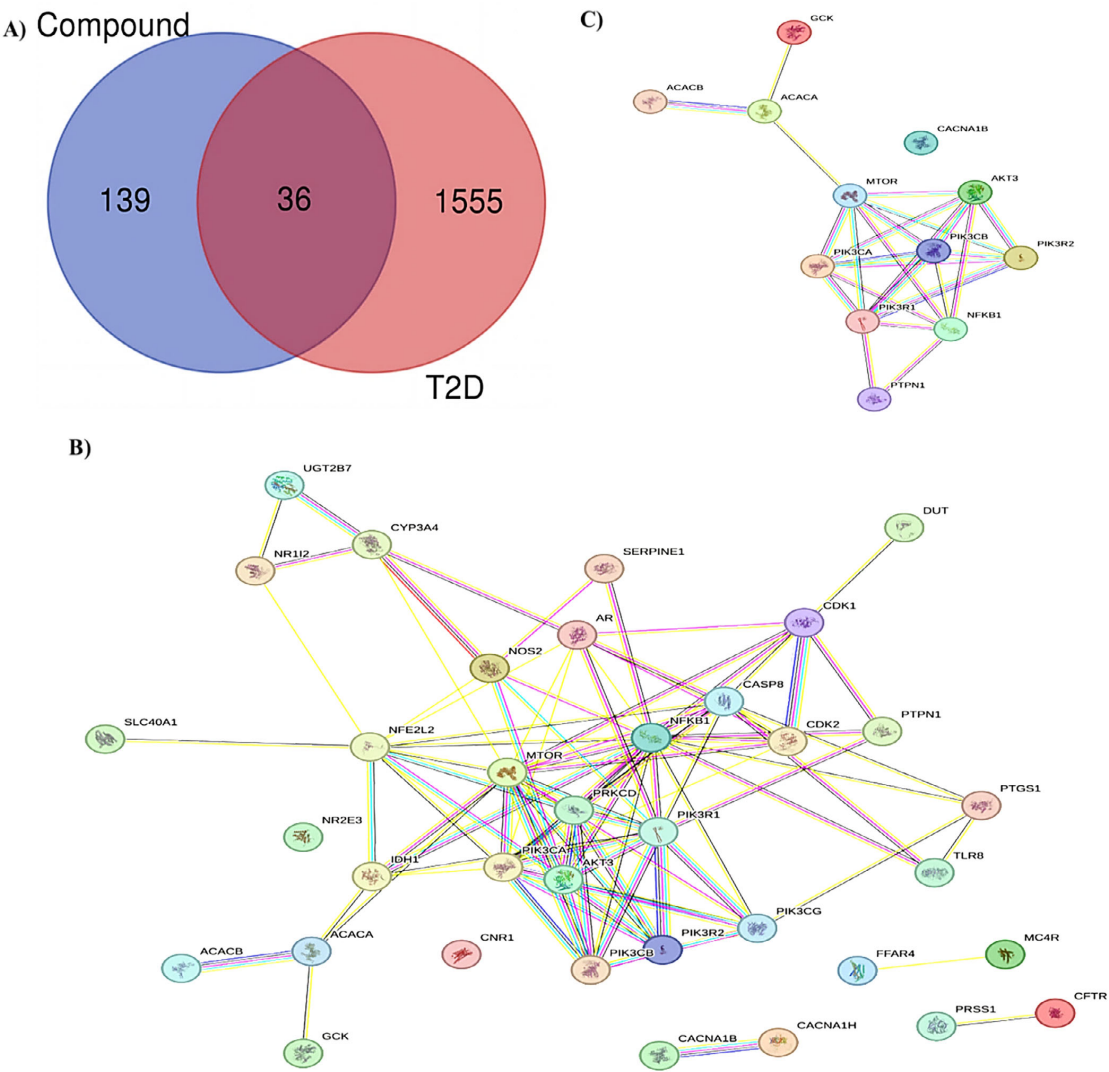
(A) Venn diagram of the interaction between compound and disease‐targeting; (B) protein–protein interaction (PPI) nodes of compound‐targets and disease‐targets using STRING 12.0; (C) PPI interaction of SN0224203 compound against T2DM.

After a supernatural library screening employing various computational analyses, the SN0224203 compound was retrieved and further subjected to network pharmacology to determine the mechanistic approach in the management of T2DM. The Superpred and Gene card database identified 175 and 15,555 targets related to the SN0224203 compound and T2DM. These targets were employed in the Venn diagram, and 36 overlapping genes were obtained, as shown in Figure 11A. Furthermore, these 36 genes were subjected to the STRING database to determine a protein–protein interaction (PPI) to analyse the functional annotation and enrichment analysis.

The STRING analysis revealed that the genes were imported into Cytoscape software. At this network intersection, network analysis was employed to determine the number of nodes in the network graph, assess their connectivity, particularly focusing on nodes with higher degrees, and identifies the biological functions they perform, as illustrated in Figure 11B. The top three most connected targets were insulin resistance (AKT3, ACACB, mTOR, NFKB1, PIK3CA, PIK3CB, PIK3R1, PIK3R2, PRKCD, PTPN1), Type 2 diabetes (CACNA1B, GCK, mTOR, PIK3CA, PIK3CB, PIK3R1, PIK3R2, PRKCD), and insulin signalling pathway (AKT3, ACACA, ACACB, GCK, mTOR, PIK3CA, PIK3CB, PIK3R1, PIK3R2, PTPN1). After removing duplicate gene nodes in the network of three targeted drug diseases. Finally, the 12 nodes of the therapeutic targets indicated that the target proteins interaction network, which is a vital play role in the interaction network target protein are located in the PPI network shows that AKT3, ACACA, ACACB, GCK, mTOR, PIK3CA, PIK3CB, PIK3R1, PIK3R2, PTPN1, CACNA1B, NFKB1, indicating that these proteins are involved in the management of T2DM as shown in Figure 11C.

#### 2.7.2 Analysis of Gene Ontology Function and Kyoto Encyclopedia of Genes and Genomes Pathway Signalling Pathway

**FIGURE 13:**
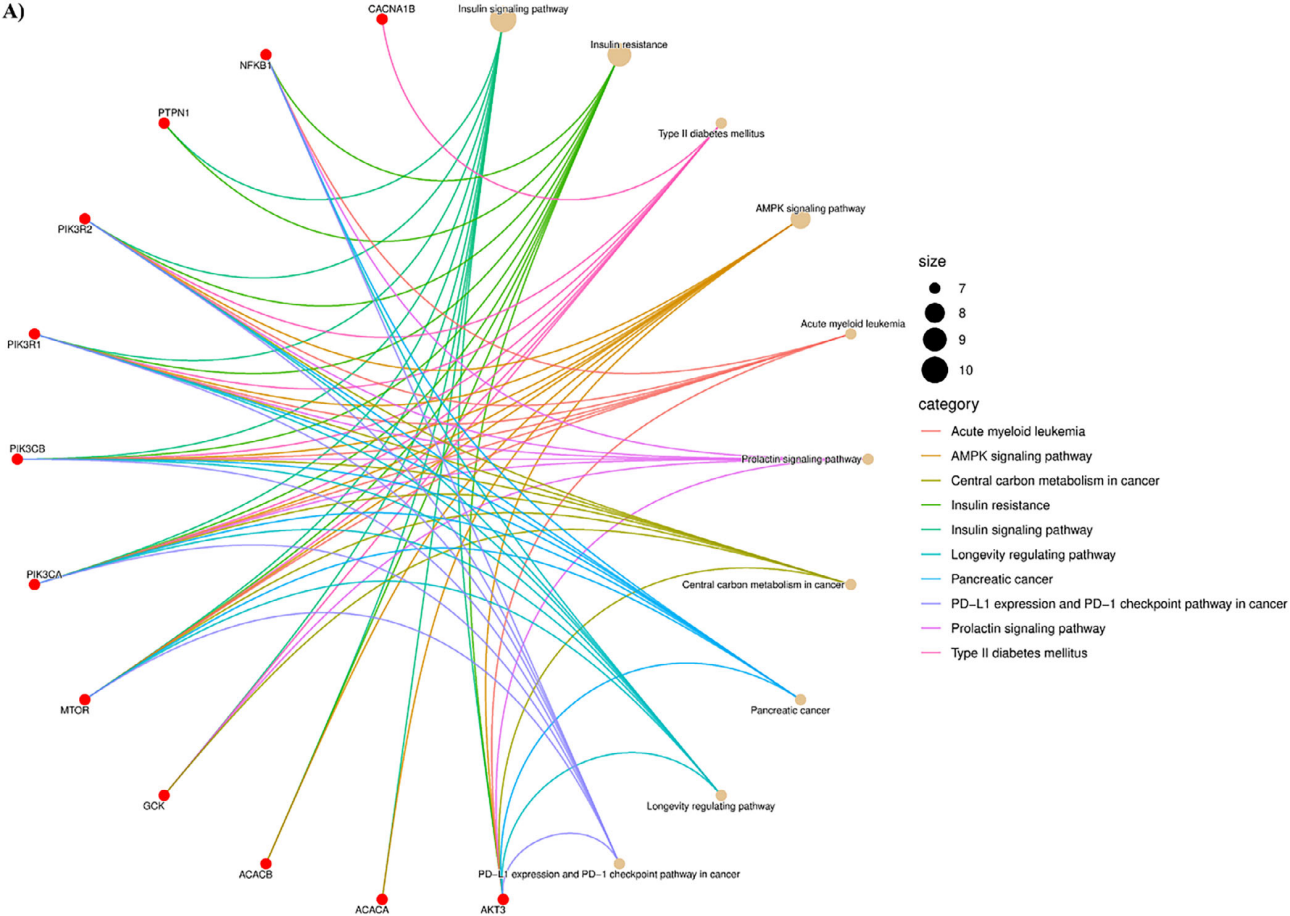
Analysis of KEGG pathway interaction; (A) the top ten pathways in the SN0224203–Type 2 diabetes target.

**FIGURE 14:**
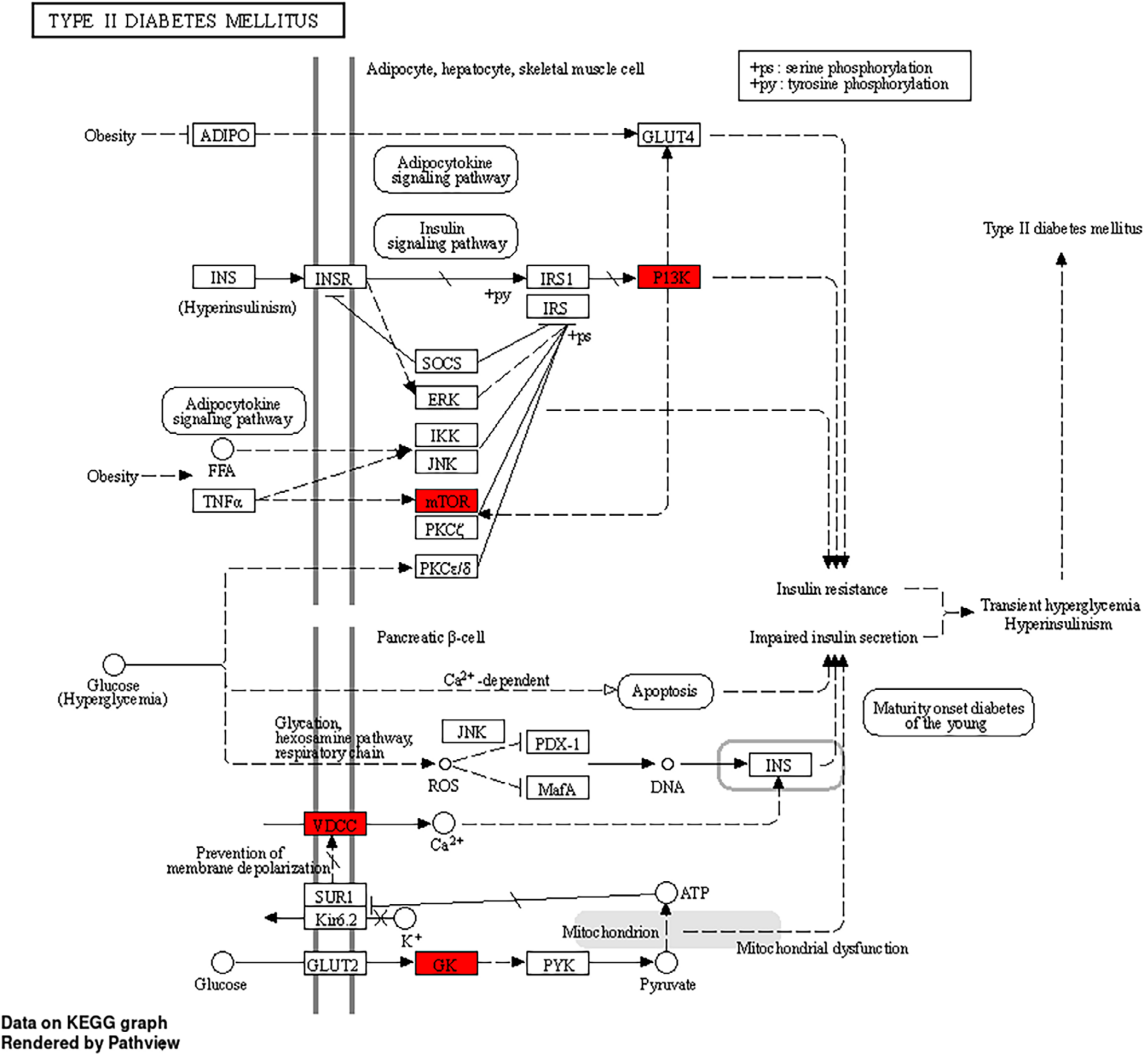
Type 2 diabetes pathway is coloured with red nodes as SN0224203 potential target proteins and white nodes are relevant potential targets in the pathway.

A total of 1162 Gene Ontology (GO) functions were retrieved from the David database, including 1046 for biological processes (BPs), 75 for molecular functions (MFs) and 41 for cellular components (CCs). We further selected the top 10 BP, CC and MF for analysis and visualization, illustrated in Table S3. The histogram represents the drug targets of GO enrichment analysis, as shown in Figure 12A–D. Furthermore, the results revealed that the BP the function of the active component of SN0224203 compound in Type 2 diabetes was focused on the lipid modification, vascular endothelial growth factor receptor signalling pathway, phosphatidylinositol phosphorylation, response to peptide hormone, response to insulin, cellular response to peptide hormone stimulus, Fc‐epsilon receptor signalling pathway, positive regulation of protein kinase B signalling, cellular response to peptide, and lipid phosphorylation. The CC are mainly included as transferase complexes, an extrinsic component of the membrane, transferring phosphorus‐containing groups, such as the mitochondrial outer membrane, phosphatidylinositol‐3‐kinase complex, TORC2 complex, organelle outer membrane, outer membrane, TOR complex, cytoplasmic side of the endoplasmic reticulum membrane, and postsynaptic cytosol. The MF includes such as receptor tyrosine kinase binding, 1‐phosphatidylinositol‐3‐kinase activity, insulin receptor substrate binding, phosphoprotein binding, protein tyrosine kinase binding, phosphatidylinositol‐3‐kinase regulator activity, phosphatidylinositol‐3‐kinase regulator activity and phosphatidylinositol phosphate kinase activity. The GO function can be used to treat the SN0224203 compound for T2DM. The Kyoto Encyclopedia of Genes and Genomes Pathway (KEGG) pathway analysis revealed that the SN0224203 compound was involved in 154 signalling pathways. The bubble in the chart visualised the top ten enriched pathways, the degree of gene enrichment was represented by abscissa, the colour depth range denoted the *p* value, and the amount of gene enrichment showed the bubble size. The three main KEGG pathways included the insulin signalling pathway, insulin resistance, T2DM, prolactin signalling pathway, AMPK signalling pathway, central carbon metabolism in cancer, pancreatic cancer, acute myeloid leukaemia, PD‐L1 expression and PD‐1 checkpoint pathway in cancer, and longevity as depicted in Figure 13A and 12E. The KEGG pathway was used to colour in the targets in the insulin signalling pathway, insulin resistance, and Type II diabetes mellitus, the targets of SN0224203–Type 2 diabetes were coloured in red and involved in the other targets in the diseases as shown in Figure 14 and Table S4.

FIGURE 12GO enrichment analysis of (A) histogram of top ten GO enrichment, (B) biological process, (C) molecular function, (D) cellular component. All nodes B, C, and D are displayed in the gradient from blue to red, showing the upregulation and downregulation of the identified genes, respectively, and (E) Enrichment of pathway analysis represents the blue to red colour of the *p* value.

**Figure:**
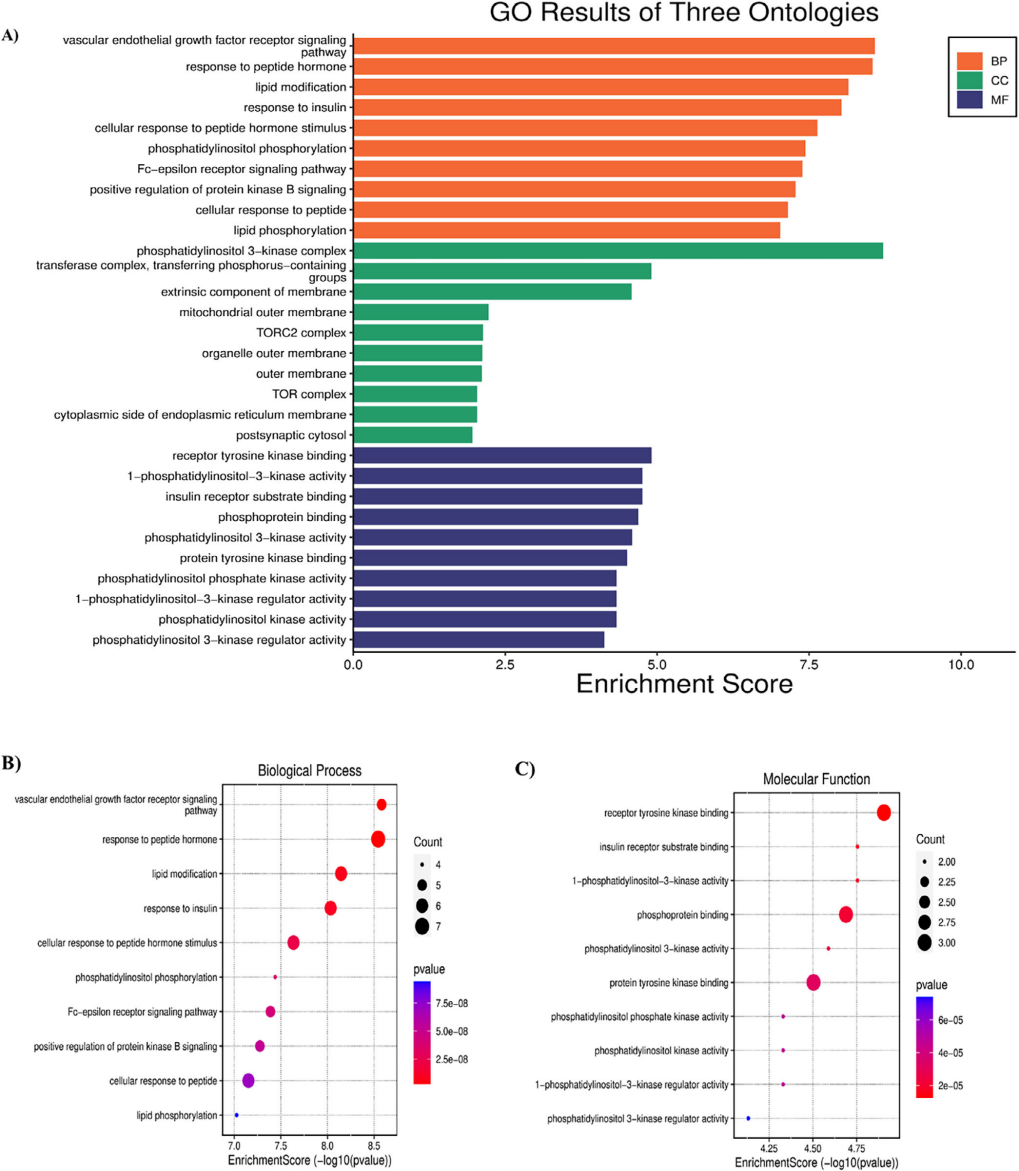

**Figure:**
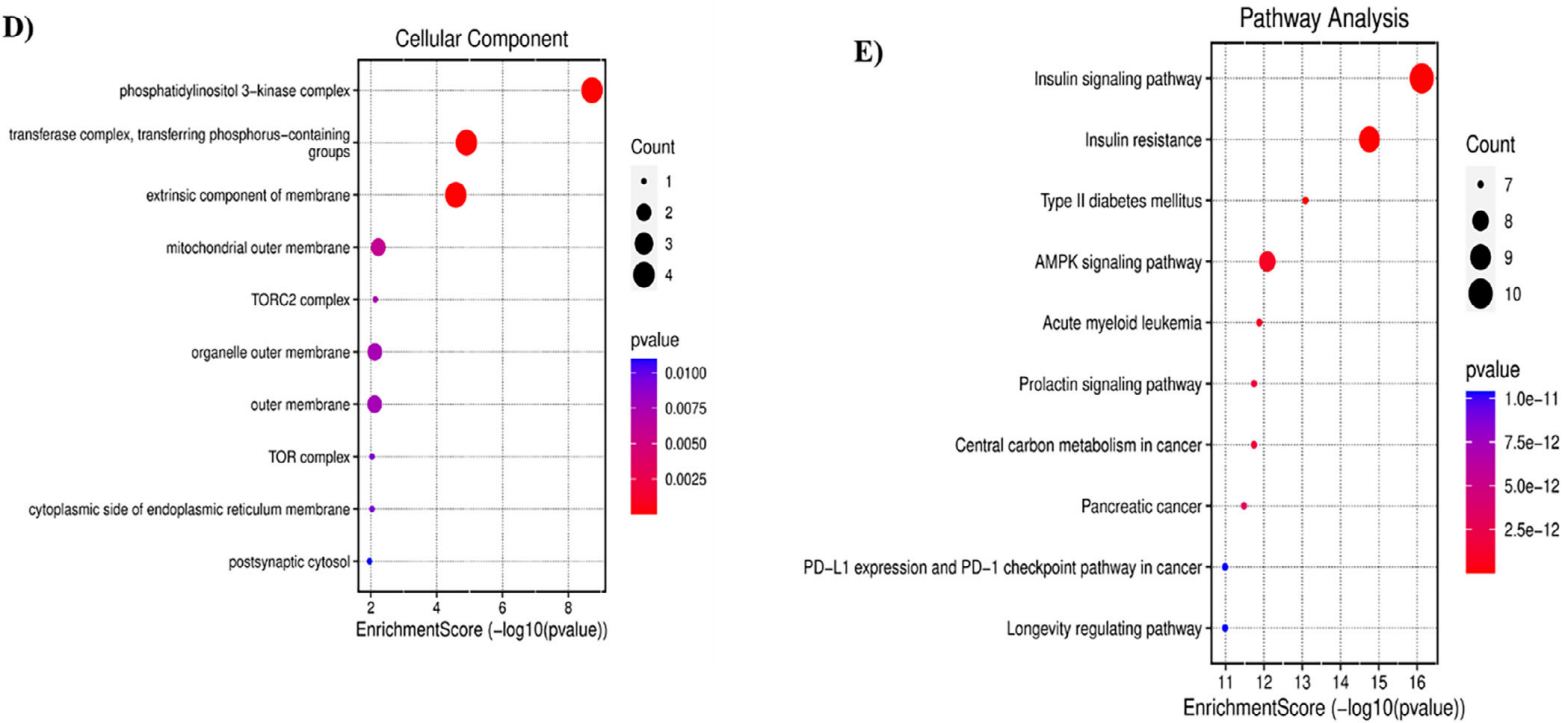

### 2.8 Structure–Activity Relationship of Natural Hit Compounds

Similarity‐based structure–activity relationship (SAR) analysis was studied on the best natural hit molecules. The natural compounds were connected in such a fashion that the bulk sugar moiety was attached to the core nucleus of the natural product, that is, the steroidal nucleus. Furthermore, the steroidal nucleus is linked to three to five aliphatic carbon linkers, accompanied by a small sugar moiety. The basic structure for all the best hit natural compounds was represented as bulk sugar moiety–core moiety–linker‐small sugar moiety.

**FIGURE 15:**
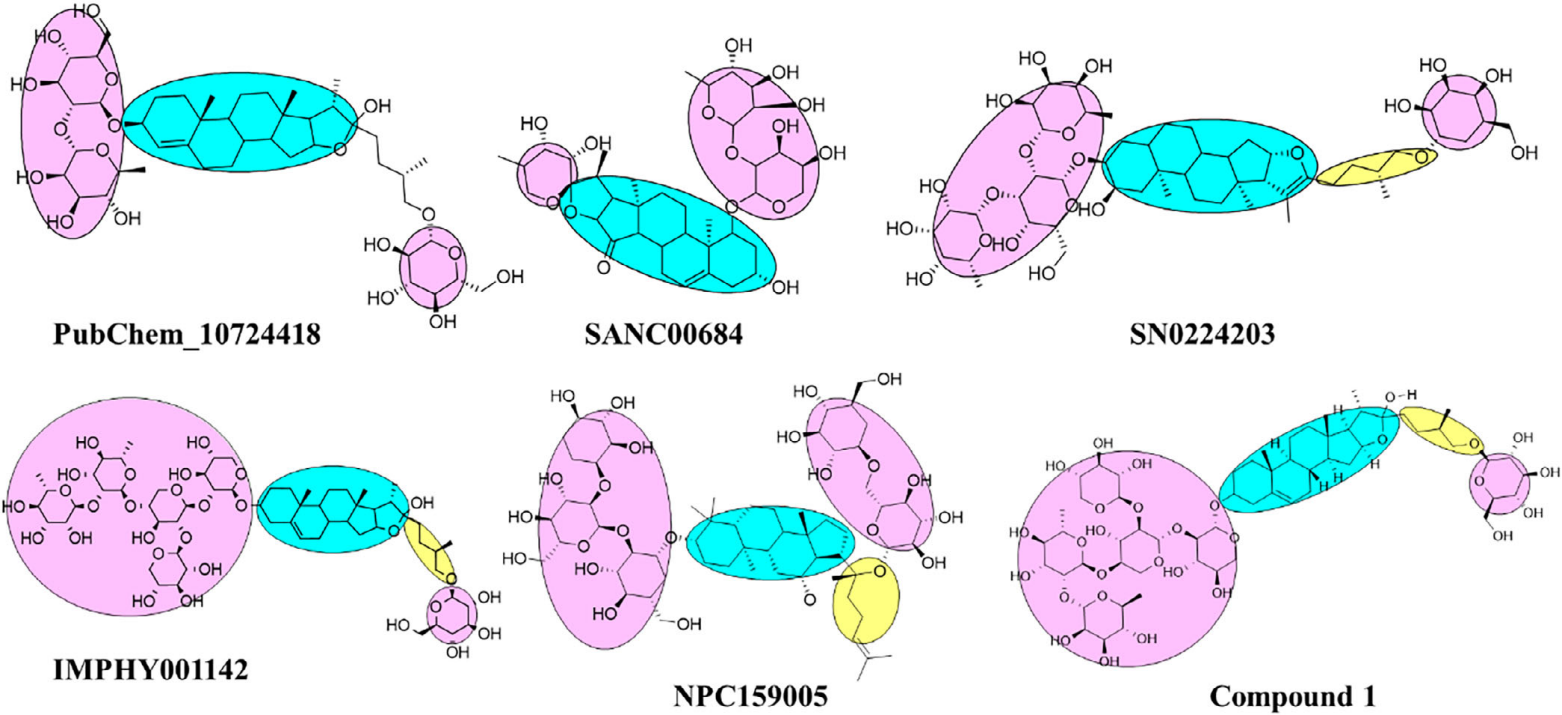
SAR analysis of best‐hit natural compounds.

Based on the docking score and binding analysis, it was observed that compounds with five sugar moieties (compound **1**) and three sugar moieties in the bulk region (SN0224203 and NPC159005) possess the best ligand to fit in the catalytic cavity of the α‐amylase enzyme. The bulk sugar moiety acts as a head region of the compound, whereas the small sugar moiety acts as a tail region. Various linkers were observed, including three‐ to five‐carbon aliphatic chains, and the ring‐constrained linkers play a crucial role in determining the size of the molecules. These computationally screened hits could be explored further as potential biological inhibitors for α‐amylase, as shown in Figure 15.

## 3 Conclusions

Diabetes mellitus is a multifactorial metabolic disorder characterised by chronic hyperglycaemia due to impaired insulin secretion, insulin resistance, or both. Its escalating global prevalence and associated long‐term microvascular and macrovascular complications underscore the urgent need for novel therapeutic interventions that are both safe and effective. The inhibition of α‐amylase, a key digestive enzyme responsible for breaking down dietary starch into glucose, represents a validated pharmacological strategy for attenuating postprandial hyperglycaemia. However, currently available α‐amylase inhibitors such as acarbose are associated with limited tolerability and gastrointestinal side effects, thereby prompting the exploration of alternative bioactive agents from natural resources.

In this study, bioactive compounds reported from BA, along with structurally similar natural product hits retrieved via Tanimoto coefficient‐based fingerprint screening, were systematically evaluated using comprehensive structure‐based computational methodologies. Molecular docking and MM‐GBSA analyses were identified. Compound **1** (Balanitesin) from BA and compound **9** (SN0224203) from the natural library are the most promising α‐amylase binders based on high‐affinity scores and favourable interaction profiles with the catalytic residues of HPA (PDB: 3BAJ). Notably, SN0224203 exhibited a superior binding energy (−128.41 kcal/mol) and docking score (−13.019 kcal/mol) compared to Balanitesin and the reference inhibitor, suggesting an enhanced inhibitory potential.

ADME and toxicity predictors indicated that although these compounds possess limited passive permeability due to their polar nature, SN0224203 demonstrated a better nontoxicity profile and acceptable drug‐likeness properties. MD simulations further confirmed the structural stability and persistent ligand‐receptor interactions of SN0224203 within the enzyme's active site over the full simulation window, reinforcing the docking observations. In addition, network pharmacology revealed that SN0224203 may influence glucose‐regulatory mechanisms through multiple gene targets, including AKT3, PIK3CA/PIK3R1, GCK, PTPN1, and mTOR, consistent with pathways relevant to T2DM pathogenesis.

Together, these computational findings identify SN0224203 as a promising multi‐targeted natural inhibitor of α‐amylase with a favourable stability profile and mechanistic relevance to diabetes‐associated pathways. Nonetheless, it is crucial to recognize that the current evidence is entirely in silico. Computational predictions alone cannot establish biological safety or therapeutic efficacy. Therefore, detailed in vitro biochemical assays, cell‐based investigations and in vivo pharmacological evaluations are imperative to substantiate the antidiabetic potential, bioavailability, metabolic stability and toxicity margins of SN0224203 before further developmental considerations.

Overall, this research provides a robust computational foundation and a prioritized candidate for future experimentation, supporting the continued exploration of plant‐derived natural molecules towards the development of improved α‐amylase inhibitors for diabetes management.

## 4 Experimental Sections

### 4.1 Identification of Similarity (Tanimoto) Approaches of Phyto Libraries

**FIGURE 16:**
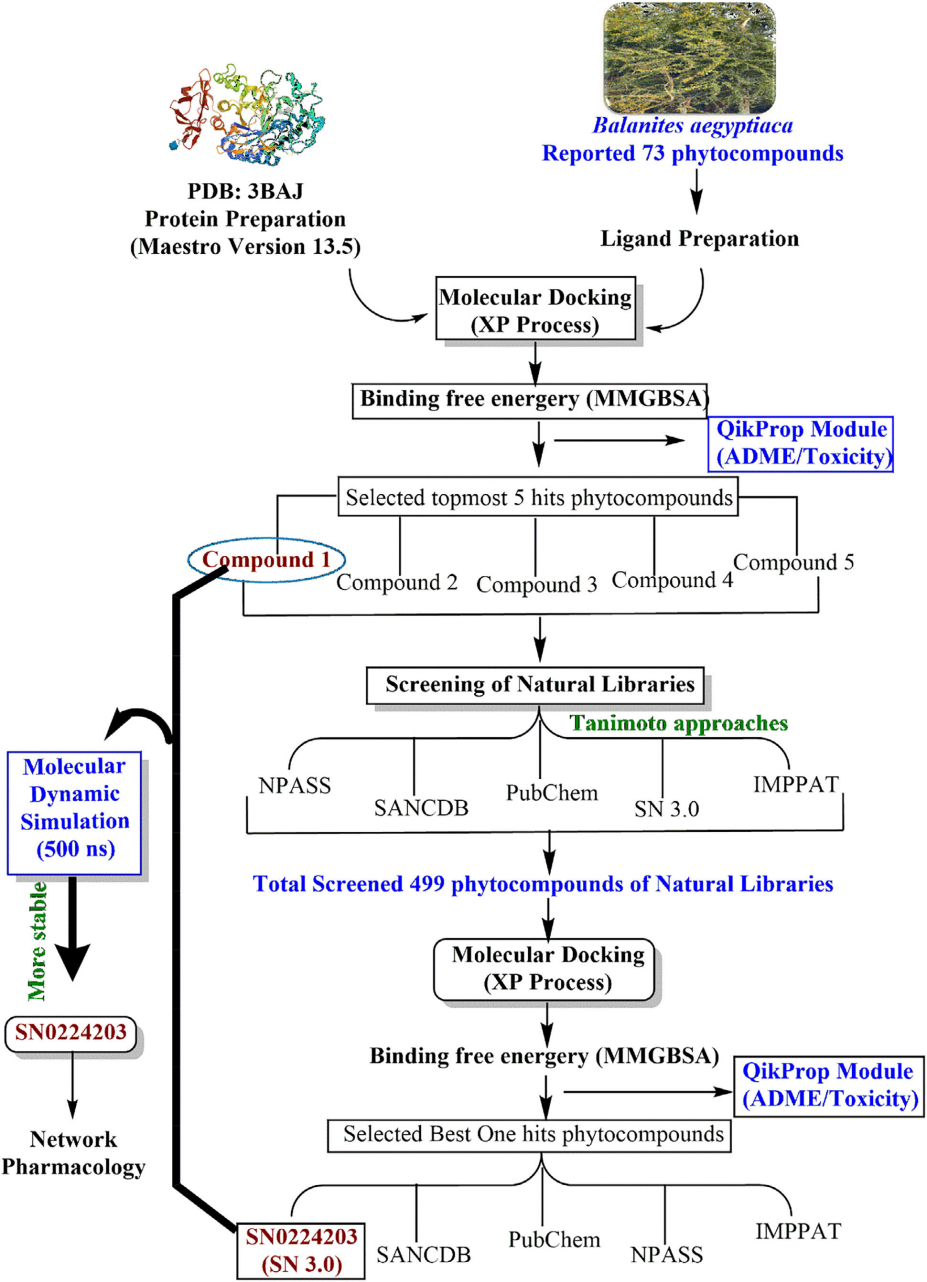
A schematic diagram of the workflow of the methodology; In the first round, all 73 reported phytocompounds were docked using the XP process, binding free energy (MM‐GBSA), and ADMET properties. The five best phytocompounds were screened for their MMGBA and ADMET properties. After completion of the first round, the five best hits phytocompounds were screened against natural libraries (NPASS, SANCDB, IMPPAT, SN 3.0, PubChem). In the second round, 499 phytocompounds from natural libraries were screened using the XP process, MM‐GBSA, and ADMET properties. Overall, the SN 3.0 library phytocompound was validated through molecular dynamics simulations (500 ns). Compound **1** and SN 3.0 (SN0224203) are validated compounds compared to the reference compound. Furthermore, SN0224203 is being evaluated for its therapeutic efficacy in managing diabetes mellitus.

The similarity search using the Tanimoto approach is used to generate a database based on the overlapping of fingerprints [59]. The Tanimoto approach applies coefficient values ranging from 0 to 1, where 1 corresponds to identical fingerprint values close to high similar molecules, whereas 0 represents a low value for similar molecules [29, 60]. The higher threshold values were used to search for similarity in the query molecules. Based on the query molecules, a similarity search was performed for the selected libraries (NPASS, SN 3.0, IMPPAT 2.0, SANCB, and PubChem) using the Tanimoto coefficient. A schematic diagram of the workflow of the methodology is represented in Figure 16.

### 4.2 Ligand Preparation

The reported 73 compounds of BA and similarity search compounds were downloaded and saved in an .sdf file [61, 62, 63]. Phytocompounds from different libraries, such as Indian Medicinal Plant, Phytochemistry and Therapeutics 2.0 (IMPPAT 2.0) https://cb.imsc.res.in/imppat/, South African Natural Compounds Database (SANCDB) https://sancdb.rubi.ru.ac.za/, Natural Products Activity and Species Sources (NPASS) https://bidd.group/NPASS/, Supernatural 3.0 (SN 3.0) https://bioinf‐applied.charite.de/supernatural_3/index.php and PubChem databases were screened for similarity search and docking studies. Ligands were prepared using the LigPrep module of the Schrodinger suite (Maestro version 13.5). Thus, the ligands were converted to 3D structures using the 2D structures by selecting features such as desalting and a physiological pH of 7 ± 2 [64].

### 4.3 Protein Preparation and Grid Generation

The x‐ray crystal structure of HPA (PDB ID: 3BAJ) was accessed from https://www.rcsb.org/. The acarbose‐derived pentasaccharide complexed with HPA was retrieved from the RCSB PDB [36]. The selection of the PDB: 3BAJ protein was based on the quality of the crystal structure, which has a 2.10 Å resolution and an R‐free value of 0.225, indicating a higher resolution and a lower R‐free value. Furthermore, the protein structure was processed using the Protein Preparation Wizard through Schrödinger's in‐built module, Maestro version 13.5 interface. Several modifications to the protein were created, including loop refinements and the addition of missing side chains using a prime module. The structures were then pre‐processed at physiological pH 7 ± 2. The review and modification option removed all unwanted heteroatoms and water molecules. Energy minimisation was applied to the Optimised Potentials for Liquid Simulations 2005 (OPLS_2005) force field, resulting in a RMSD of 0.30 Å. Furthermore, the grid was generated in the protein active sites using a 3D grid box size with dimensions of (20 Å × 20 Å × 20 Å) using Maestro version 13.5 [65, 66].

### 4.4 Molecular Docking

#### 4.4.1 Validation Protocol

The validated protocol used Maestro version 13.5 (Schrodinger software 2023‐1 suite). The standard compound of ligand and co‐crystal ligand was superimposed. The RMSD value and docking score of the superimposed structure were obtained.

#### 4.4.2 Molecular Docking of Phytocompounds From BA

The reported phytocompounds were studied for molecular docking using the XP mode. Further topmost hit molecules were analysed using the molecular mechanics‐generalised born surface model (MM‐GBSA) to calculate the binding energies of protein–ligand complexes using Schrodinger software 2023‐1 (Maestro version 13.5) [67].

#### 4.4.3 Molecular Docking of Natural Libraries

The topmost compounds from the BA plant were used for a similarity search using the Tanimoto approach across different natural libraries, including SANCDB, NPASS, SN 3.0, IMPPAT and the PubChem database, to identify phytocompounds that inhibit α‐amylase. Phytocompounds were docked using the XP mode to obtain the topmost hit molecules [68, 69].

#### 4.4.4 Estimation of ∆*G* Energy Using MM‐GBSA

The MM‐GBSA was used to calculate the ∆*G* energy of protein–ligand complexes. MM‐GBSA of all‐natural libraries and BA compounds was implemented using the Prime module in the Schrödinger Suite version 2023‐1. The binding energies was calculated by the implicit solvation model, that is, variable‐dielectric generalised Born model (VSGB) with OPLS_2005 force field and keeping the residues within 5 Å distance from the ligand using Maestro version 13.5 (Schrodinger 2023‐1) [70].

#### 4.4.5 Determination of In Silico ADME and Toxicity

In silico pharmacokinetic studies (ADME) were carried out using the Qikprop module version 4.9. Various physicochemical parameters was calculated, such as CNS activity, PISA, WPSA, log BB, apparent Caco‐2 permeability (PCaco‐2), aqueous solubility (log *S*), confirmation‐independent aqueous solubility (Cl log *S*), percentage of HOA, MDCK, Skin permeability (log *K*_p_), HOA, rule of five and rule of three [71]. Webserver‐based tools for toxicity studies were also analysed using the ProTox‐II programme for best‐hit molecules.

### 4.5 Determination of Network Pharmacology

#### 4.5.1 Screening of Bioactive Compounds and Construction of Active Component Targets

After molecular docking, the best hits are used in the Superpred online database: https://prediction.charite.de/subpages/target_prediction.php. The target gene names were obtained using the UniProt online database https://www.uniprot.org/ with the *H. sapiens* species [72]. The targeted compounds and their targets were constructed, and duplicates were removed. The targets related to T2DM were retrieved from the Human Gene Database (https://www.genecards.org/). Compounds and T2DM‐related targets were imported into the Venn diagram tool to isolate the common targets https://bioinformatics.psb.ugent.be/webtools/Venn/ for analysis [73].

#### 4.5.2 Construction of PPI, GO Function and KEGG Analysis

The active component of a compound and its intersection points with disease targets were identified using the STRING database, version 12.0 (https://string‐db.org/), with a focus on human PPIs. A PPI network was constructed with default settings for the species ‘*H. sapiens*’ [74]. Using STRING version 12.0, the active compounds and T2DM were identified and further analysed, visualising the network's interaction using Cytoscape 3.10.2 software https://cytoscape.org/, and counting the nodes in the network. The active compounds associated with T2DM regulation were added to the GO online platform, where they underwent enrichment analysis for MFs, BPs and CCs. The results were visualised using the biological resources tool [75]. The enrichment analysis of KEGG pathways was performed utilising the Cytoscape software version 3.10.2 [76].

### 4.6 MD Simulation

The MD simulations were performed to assess the stabilities of the P‐L complexes for 500 ns using Desmond software in the Schrödinger Suite 2023‐1 [77]. The simulation was performed in three stages: system building, minimisation and MD. The P‐L complex was prepared using the system builder. The MD study of the natural compounds was conducted using an orthorhombic box (10 Å × 10 Å × 10 Å), and the SPC solvent model was employed with periodic boundary conditions. Furthermore, Na^+^/Cl^−^ ions were added to all the complexes to neutralise and stabilise the system. A neutral system solvated within the OPLS2005 force field was then subjected to an unconstrained energy minimisation process, employing the steepest descent method to resolve steric clashes. During the energy minimisation, the ensembles used were NPT. The pressure of 1 bar and a temperature of 300 K were maintained using a Nose–Hoover thermostat [78] and a Martyna–Tuckerman–Klein barostat [79]. The trajectories were measured at intervals of 1.2 and 10 ps. Long‐range electrostatic interactions were calculated using the smooth particle mesh Ewald method with a precision of 1e−09, whereas short‐range Van der Waals and Coulomb interactions were approximated by applying a 9.0 Å cut‐off radius.

## 5 Limitations of the Study

Although this study offers promising insights into the potential of BA phytochemicals as α‐amylase inhibitors through computational approaches, it is important to recognise its limitations. All analyses were conducted in silico using molecular docking, dynamic simulations, ADME/toxicity predictions, and network pharmacology. While these methods are valuable for early‐stage screening and hypothesis generation, they cannot fully substitute for experimental validation.

One key limitation is the lack of in vitro and in vivo studies to confirm the biological activity, pharmacokinetics, and safety profiles of the selected compounds, particularly SN0224203. Predictions related to ADME, and toxicity are based on models that may not account for the complex interactions within living organisms. Moreover, although network pharmacology provides a systems‐level understanding of possible target interactions, it does not reflect the dynamic changes and compensatory mechanisms present in biological systems.

Another possible limitation is the inherent variability in phytochemical content, which depends on the plant source, growth conditions, and extraction methods, and may influence reproducibility. Lastly, while the computational models used are state‐of‐the‐art, their accuracy depends on the quality of structural and biological input data.

Therefore, while this work lays a solid foundation for identifying potential antidiabetic agents from BA, future experimental studies are essential to validate these findings and assess their therapeutic relevance in a real‐world setting.

## Author Contributions

**Surendra Kumar Gautam**: data collection, primary draft, and compilation. **Rakesh Kumar Paul**: experimentation, primary draft validation, and upgradation. **Smita Jain**: network pharmacology, review, and upgradation. **Iqrar Ahmad**: analysis of molecular simulation and validation. **Ammar A. Razzak Mahmood**: analysis of molecular simulation and validation. **Harun Patel**: analysis of molecular simulation and validation. **Penke Vijaya Babu**: experimentation and validation. **Muhammad Wahajuddin**: conceptualisation, resource management, proofreading, and supervision. **Kaisar Raza**: conceptualisation, resource management, proofreading, and supervision.

## Conflicts of Interest

The authors declare no conflicts of interest.

## Supporting information




**Supporting File 1**: cbdv70906‐sup‐0001‐SuppMat.docx

## Data Availability

The data that support the findings of this study are available from the corresponding author upon reasonable request.
