# Supplementary material for: Computational Analysis of Balanites aegyptiaca Phytochemicals as Inhibitors of Human Pancreatic α‐Amylase
Source: Chem Biodivers. 2026 Feb 12;23(2):e03133. doi: 10.1002/cbdv.202503133 (PMC12895220; doi:10.1002/cbdv.202503133)
Supplement: Supplementary file 1 — Supporting File 1: cbdv70906‐sup‐0001‐SuppMat.docx [file CBDV-23-e03133-s001.docx]

**Computational Analysis of *Balanites aegyptiaca* Phytochemicals as Inhibitors of Human Pancreatic α-Amylase**

Surendra Kumar Gautam^1^, Rakesh Kumar Paul^1^, Smita Jain^2^, Iqrar Ahmad^3,4^, Ammar A. Razzak Mahmood^5^, Harun Patel^3^, Penke Vijaya Babu^6^, Muhammad Wahajuddin^7*^, Kaisar Raza^1,7*^,

^1^ Department of Pharmacy; School of Chemical Sciences and Pharmacy; Central University of Rajasthan, NH-08 Bandarsindri, Ajmer, Rajasthan, 305 817, India

^2^ Department of Pharmacology, School of Pharmacy and Technology Management, SVKM’s NMIMS Deemed-to-be University, Shirpur, Maharashtra, 425 405, India

^3^Department of Pharmaceutical Chemistry, R. C. Patel Institute of Pharmaceutical Education and Research, Shirpur, Maharashtra, 425 405, India

^4^ Department of Pharmaceutical Chemistry, Prof. Ravindra Nikam College of Pharmacy, Gondur, Dhule, Maharashtra, 424 002, India

^5^ Department of Pharmaceutical Chemistry, University of Baghdad, Bab-AL-Mouadam, Baghdad, Iraq

^6^ Department of Pharmaceutical Sciences, Tikvah Pharma Solutions Pvt Ltd, IDA-Cherlapally, Hyderabad, Telangana, 500 051, India

^7^ Institute of Cancer Therapeutics, School of Pharmacy and Medical Sciences, University of Bradford, BD7 1DP, United Kingdom

***Corresponding Authors**

Dr Muhammad Wahajuddin

Institute of Cancer Therapeutics, School of Pharmacy and Medical Sciences, University of Bradford, BD7 1DP, United Kingdom, m.wahajuddin@bradford.ac.uk

Dr Kaisar Raza

Department of Pharmacy, School of Chemical Sciences and Pharmacy, Central University of Rajasthan, Bandarsindri, Dist. Ajmer, Rajasthan-305817, India, drkaisar@curaj.ac.in

Institute of Cancer Therapeutics, School of Pharmacy and Medical Sciences, University of Bradford, BD7 1DP, United Kingdom, k.raza6@bradford.ac.uk

**Supplymentry informations**

**Figures**


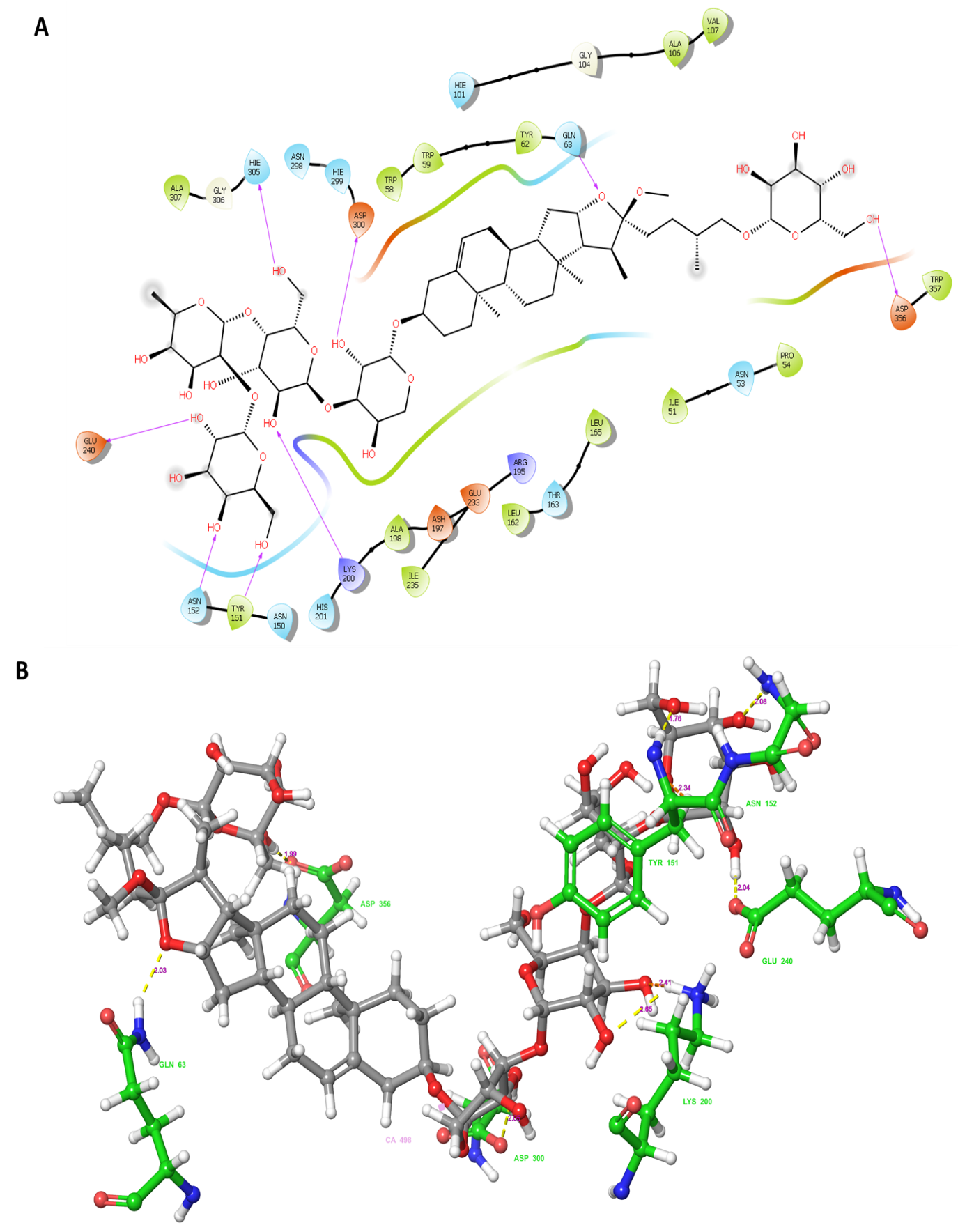


**Figure. S1.** 2D (A) and 3D (B) P-L complexes of Compound 2 with 3BAJ protein


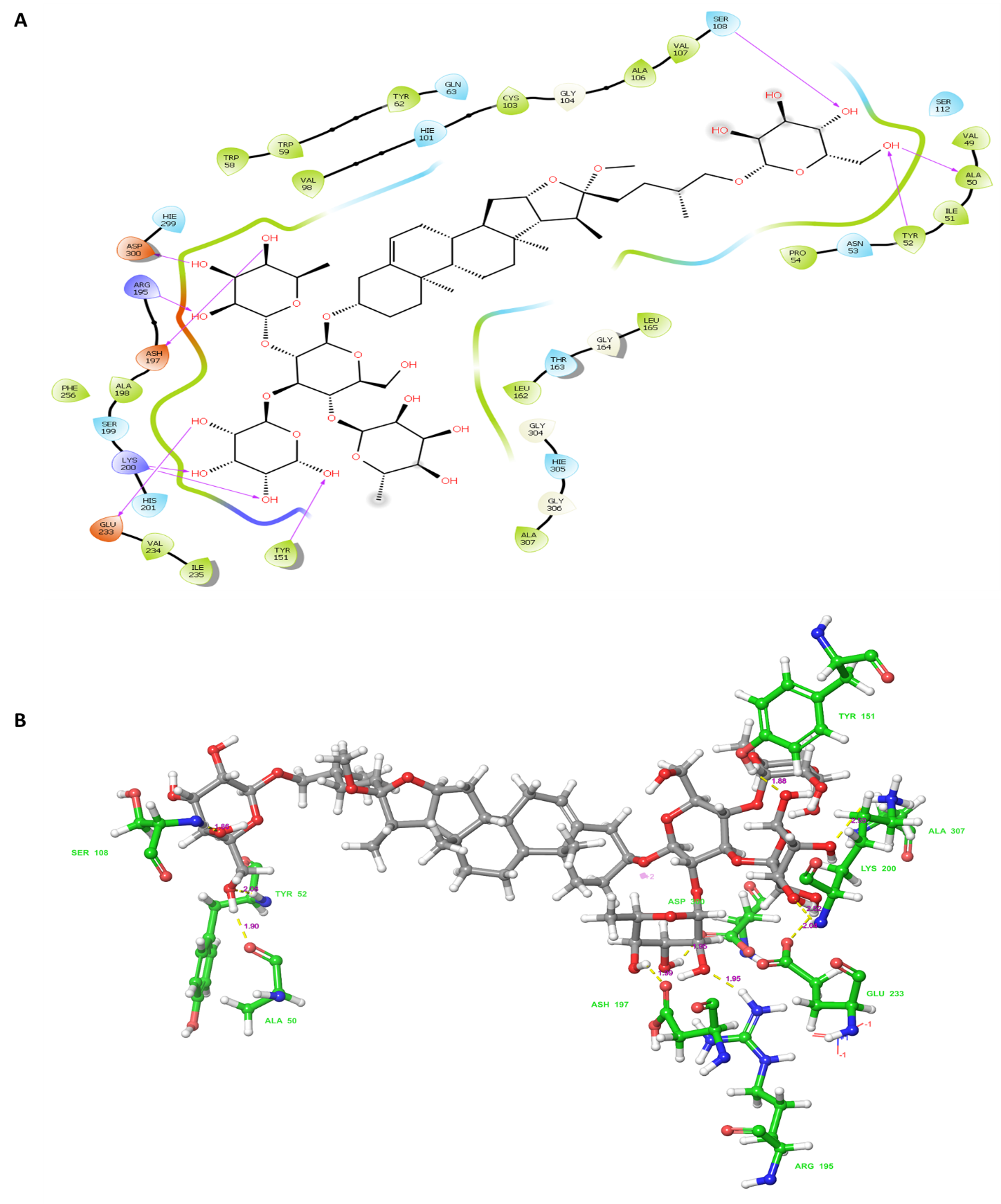


**Figure. S2.** 2D (A) and 3D (B) P-L complexes of Compound 3 with 3BAJ protein


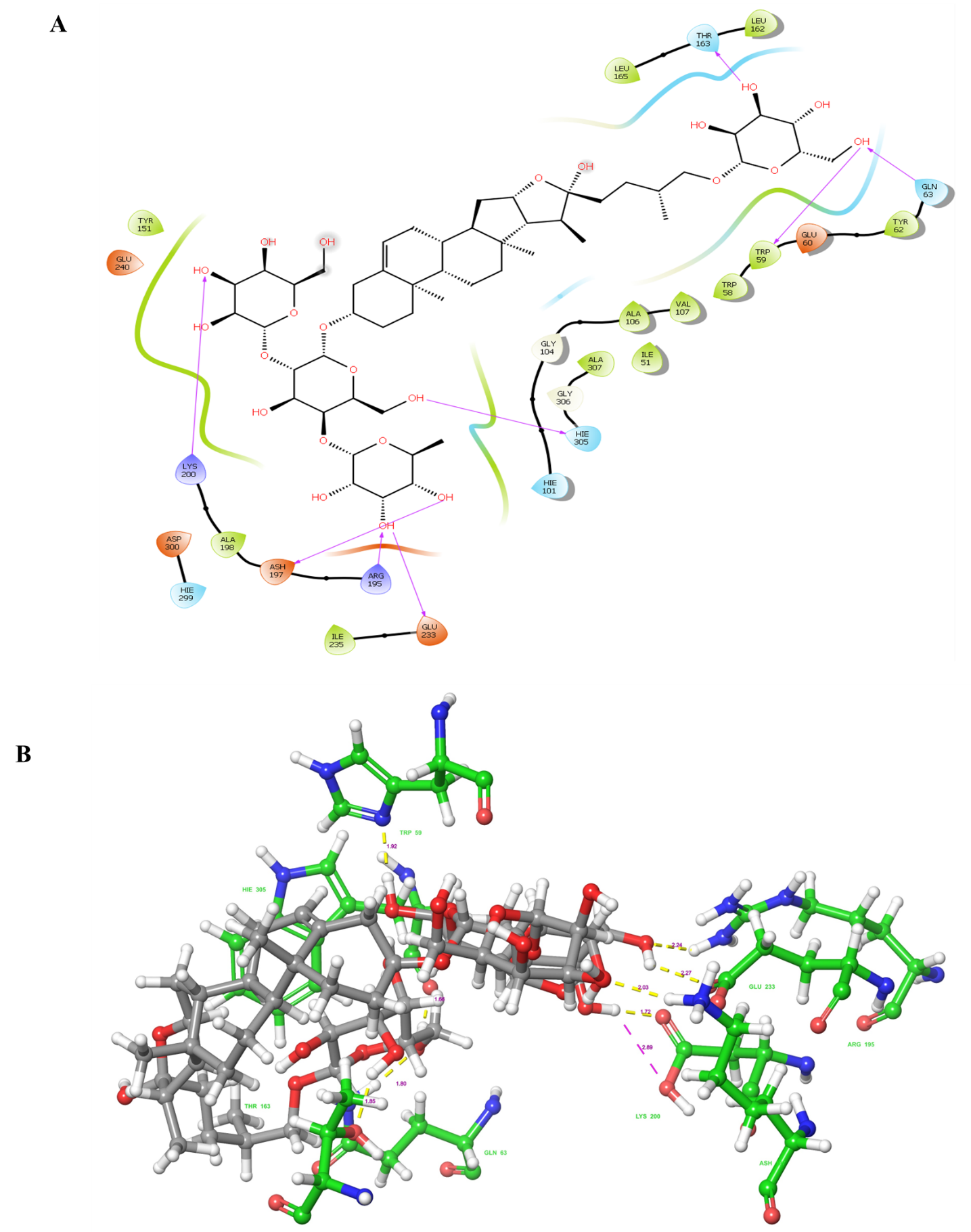


**Figure. S3.** 2D (A) and 3D (B) P-L complexes of Compound 4 with 3BAJ protein

**
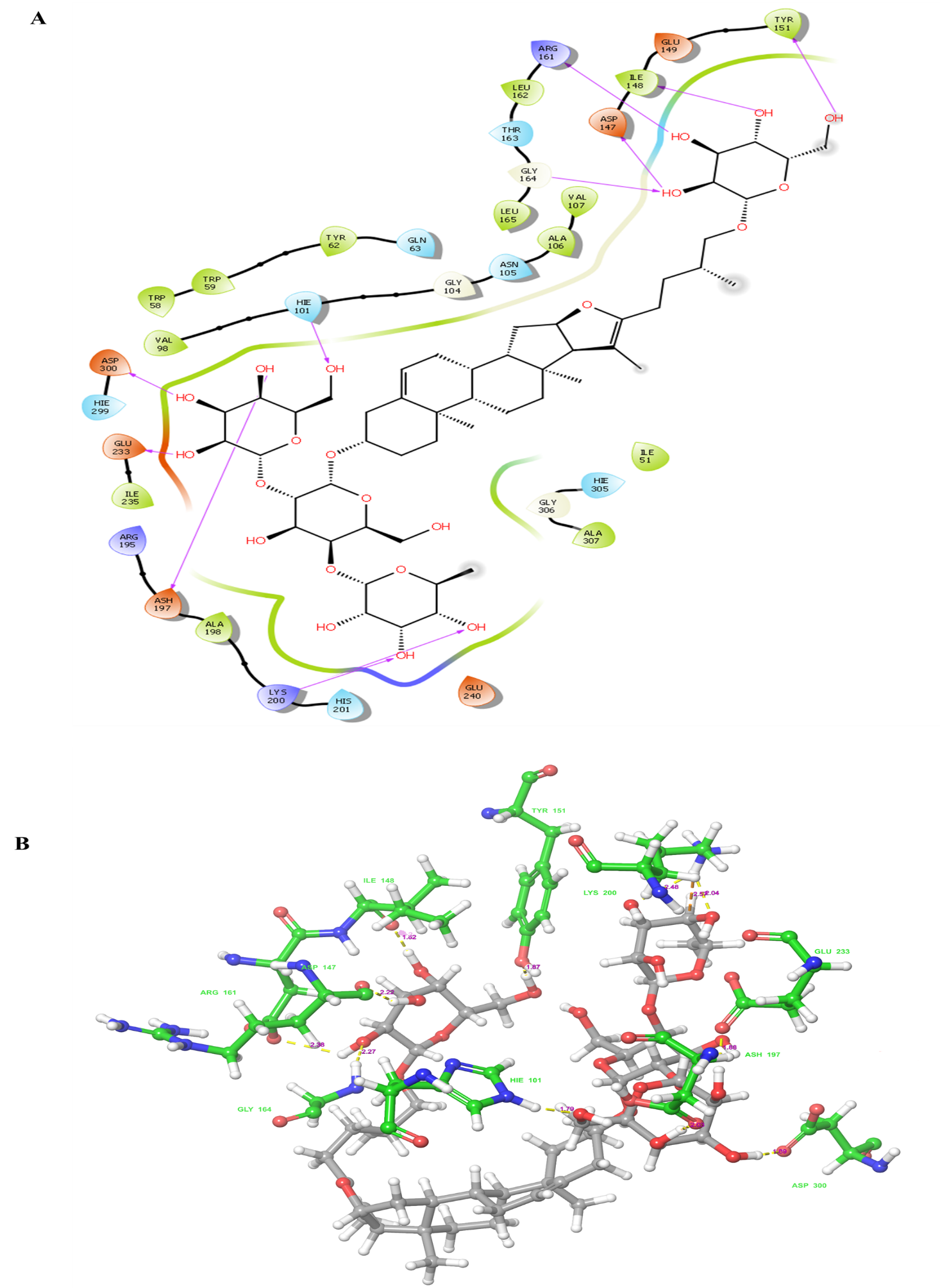
**

**Figure. S4.** 2D (A) and 3D (B) P-L complexes of Compound 5 with 3BAJ protein


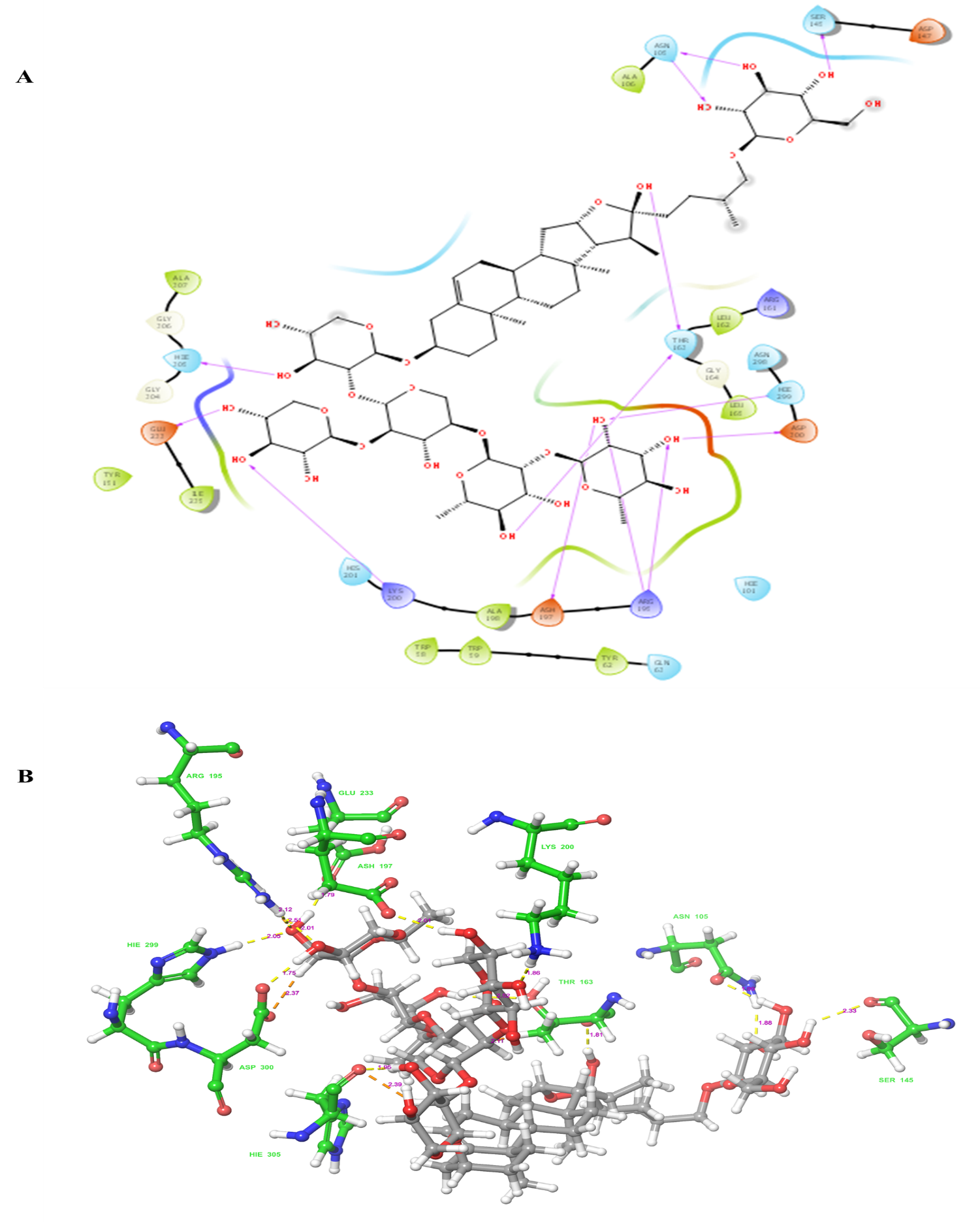


**Figure. S5.** 2D (A) and 3D (B) P-L complexes of Compound 6 (IMPHY001142) with 3BAJ protein


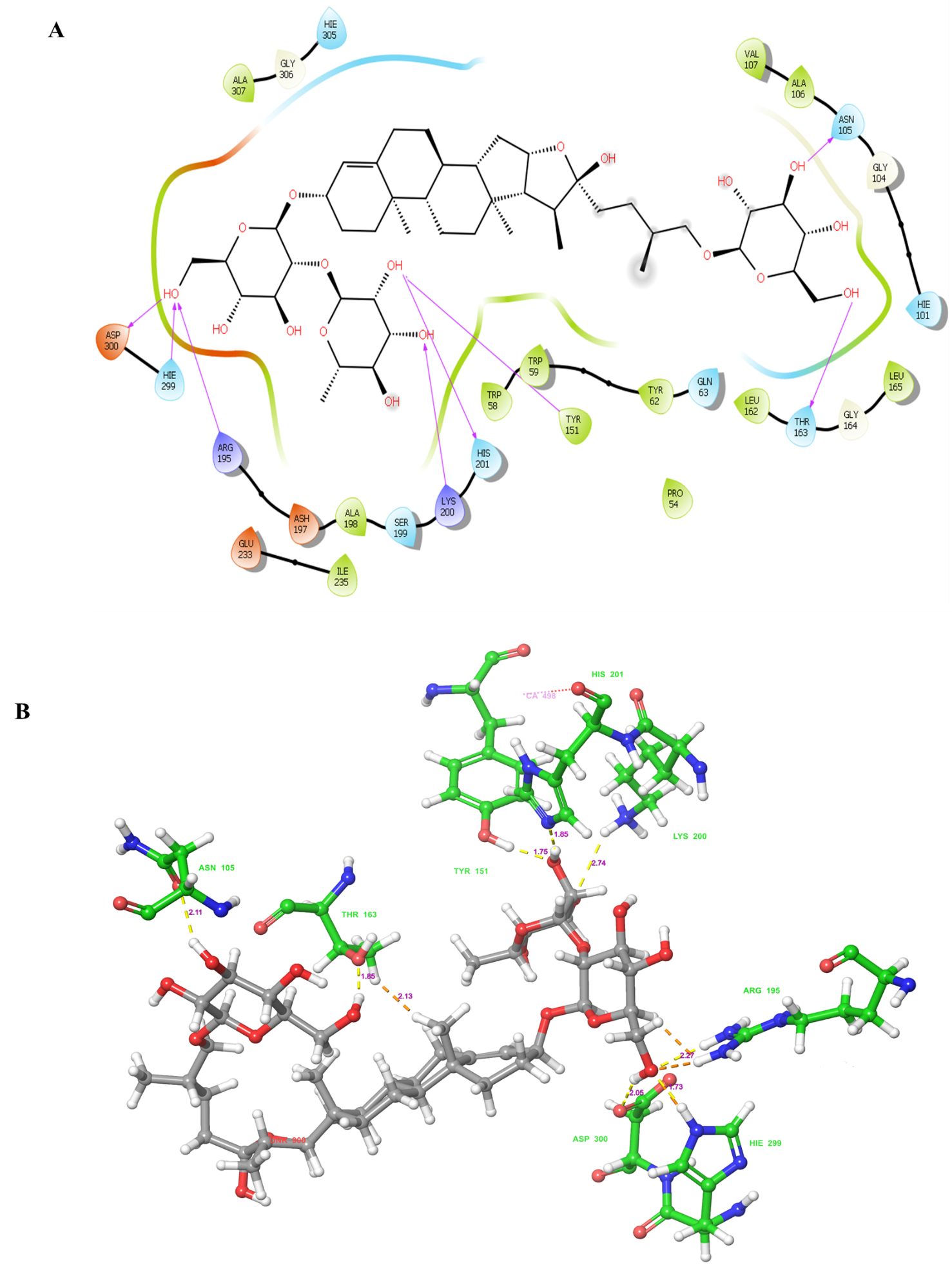


**Figure. S6.** 2D (A) and 3D (B) P-L complexes of Compound 7 (10724418) with 3BAJ protein


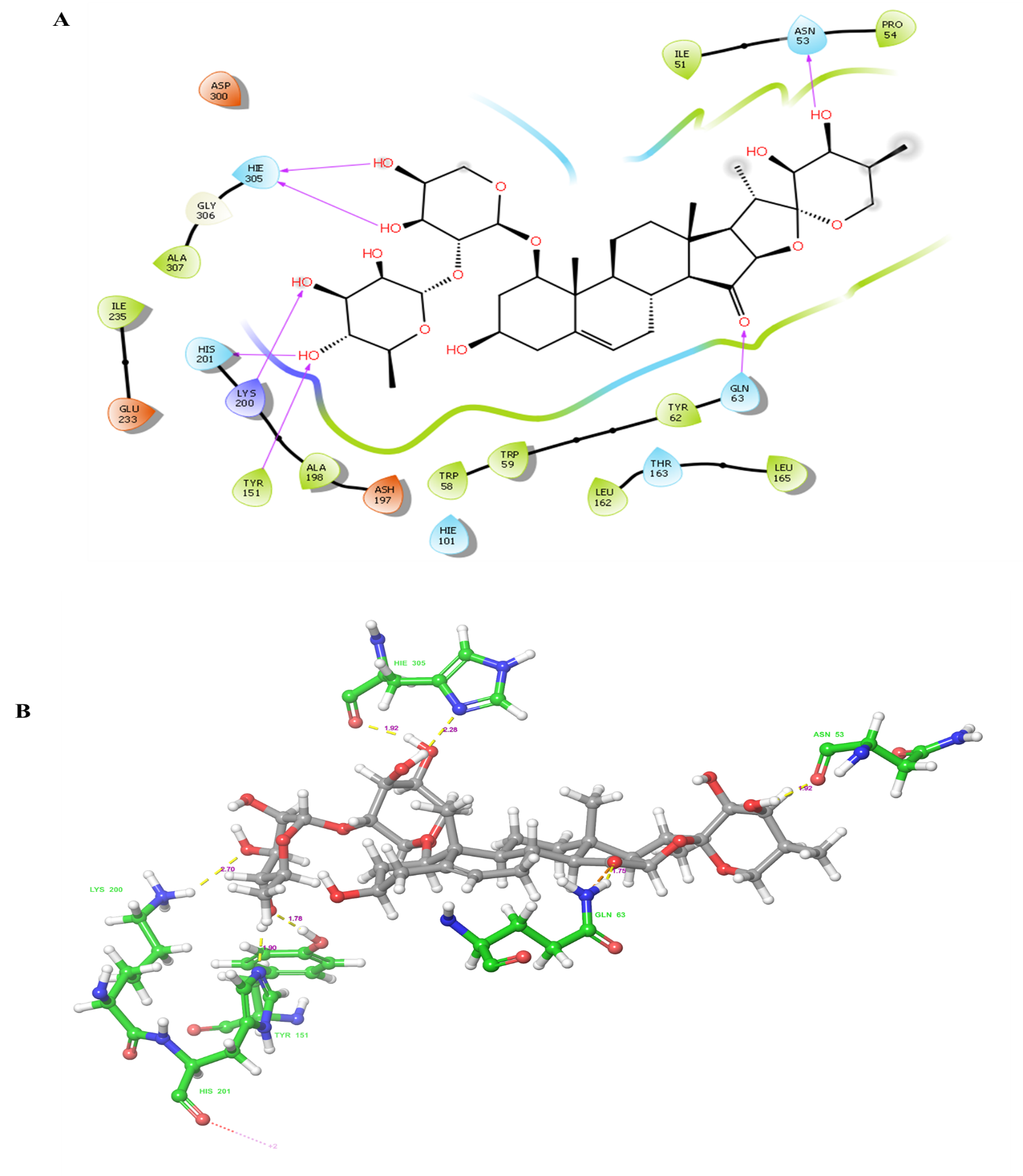


**Figure. S7.** 2D (A) and 3D (B) P-L complexes of Compound 8 (SANC00684) with 3BAJ protein


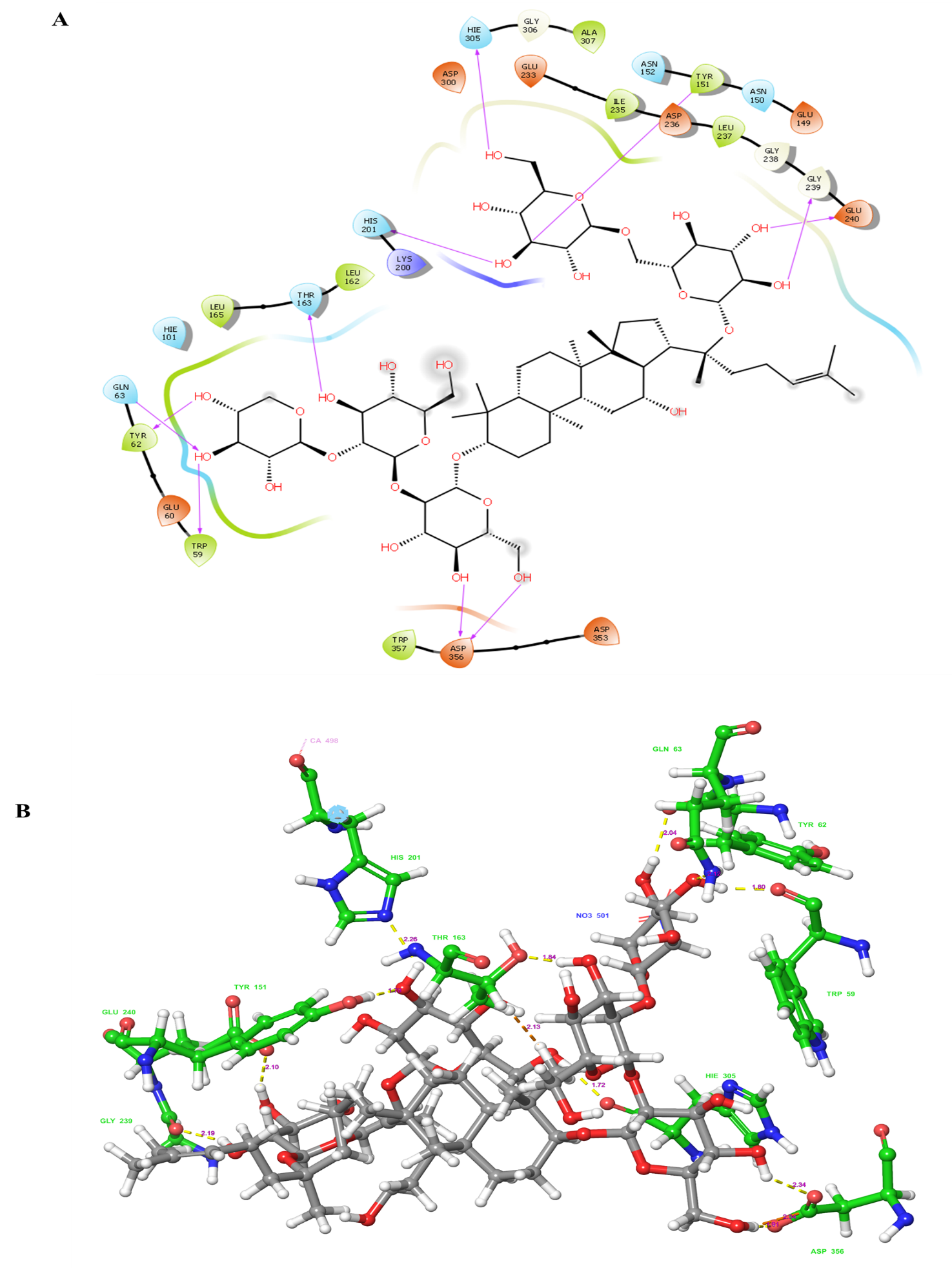


**Figure. S8.** 2D (A) and 3D (B) P-L complexes of Compound 10 (NPC159005) with 3BAJ protein


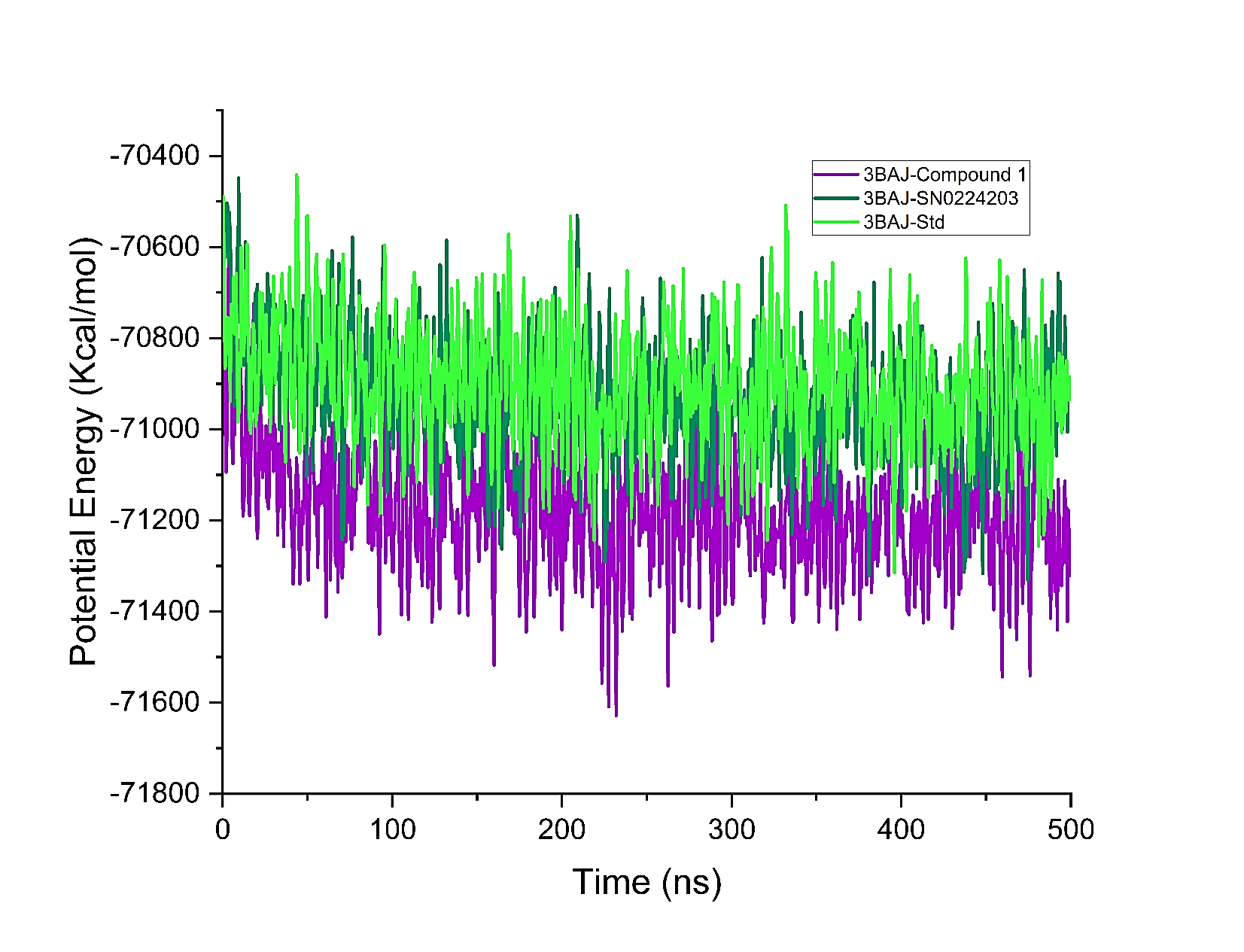


**Figure. S9**. Time dependant Potential energy analysis of 3BAJ-Compound 1, 3BAJ-SN0224203, and 3BAJ-standard complexes

**
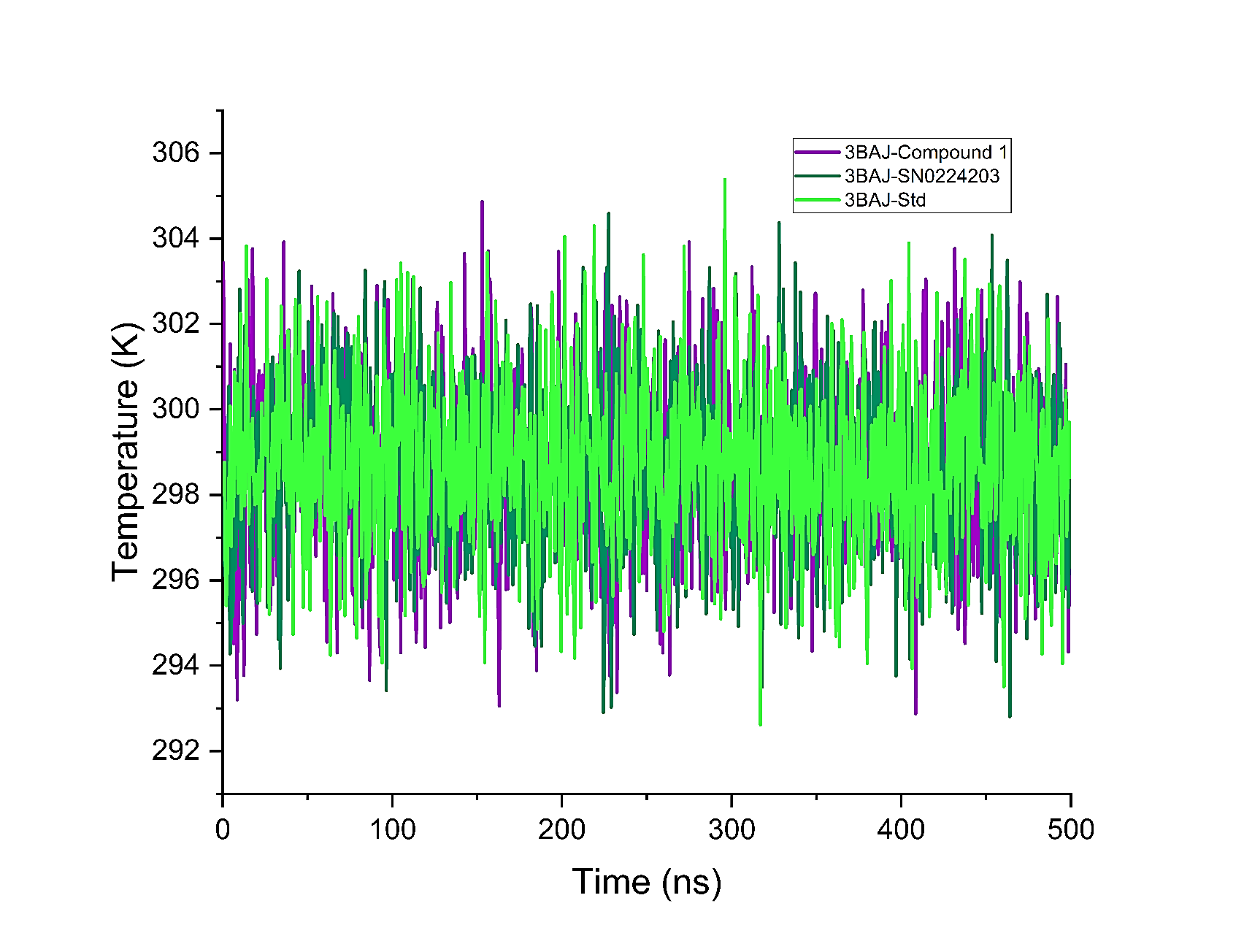
**

**Figure. S10**. Time dependant temperature analysis of 3BAJ-Compound 1, 3BAJ-SN0224203, and 3BAJ-standard complexes

**

**

**Figure S11. Correlation graph of ligands using the known affinity capacity of MMGBSA and pKd/Ki values of compounds.**

**
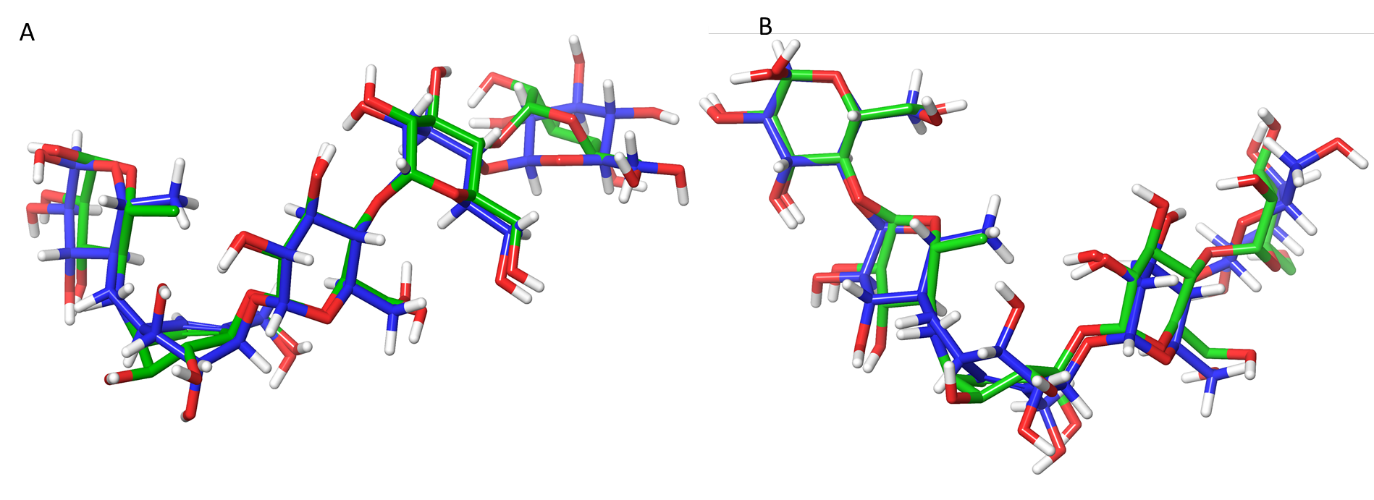
**

**Figure S12. Superimpositions of A)** ligand of structures (green colour) and docked pose of co-crystal ligand (blue colour) of PDB: 2QV4; **B)** posed of co-crystal ligand structures of PDB: 1XD0 (blue colour-docked co-crystal ligand, green colour-crystal ligand structure) **.**

**Table S1.** Physiochemical properties of the reported compound and natural library of docking scores, minimization of binding energy (∆G) and interaction of Protein Data Bank (PDB: 3BAJ) with ligand interactions

| **S.No.** | **Compounds** | **Structures** | **Dock Score** | **∆G bind** | **P-L interactions (H- H-bonds)** |
| --- | --- | --- | --- | --- | --- |
| **Reported Compounds of BA** | | | | | |
|  | **Compound 2** |  | -10.929 | -128.889 | Gln63, Asp356, Hie305, Tyr151, Asn152, Glu240, Lys200, Asp300 |
|  | **Compound 3** |  | -11.353 | -166.261 | Ala50, Tyr52, Ser108, Ala307, Glu233, Tyr151, Arg195, Asp300, Lys200, Ash197 |
|  | **Compound 4** |  | -11.797 | -100.808 | Hie305, Thr163, Gln63, Trp59, Lys200, Ash197, Arg195, Glu233 |
|  | **Compound 5** |  | -11.323 | -116.779 | Lys200, Glu233, Asp300, Ash197, Hie101, Gly164, Asp147, Arg161, Ile148, Tyr151 |
| **Natural Libraries Compounds** | | | | | |
|  | **Compound 6 (IMPHY001142)** |  | -12.864 | -100.31 | Hie305, Asp300, Hie299, Arg195, Arg195, Ser145, Asn197, Lys200, Glu233, Thr163, Thr163, Asn105, Asn105 |
|  | **Compound 7 (10724418)** |  | -10.730 | -114.73 | Asn105, Thr163, Asp300, Hie299, Arg195, Lys200, His201, Tyr151 |
|  | **Compound 8 (SANC00684)** |  | -11.372 | -105.05 | Hie305, Asn53, Gln63, Lys200, Tyr151, His201 |
|  | **Compound 10 (NPC159005)** |  | -13.262 | -119.29 | Hie305, His201, Tyr151, Asp356, Asp356, Gln63, Tyr62, Trp59, Thr163, Glu240, Gly239 |

**Table S2.** Physiochemical properties (ADME) and Toxicity with standard limits of selected ligand obtained from the docking studies.

| **S.No.** | **Phytochemicals** | **CNS (-2 inactive/**  **+2active)** | **PISA (0-450)** | **WPSA (0-175)** | **logS (-6-0.5)** | **Cl logS (-6.5-0.5)** | **log BB (-3-1.2)** | **PCaco (<25poor,**  **>500 great)** | **PMDCK (<25 poor, >500 great)** | **logKp (-8.0- -1.0)** | **HOA (1, 2, 3)** | **%HOA (>80 high, <25 poor)** | **ROF (max 4)** | **ROT (max 3)** | **Predicted Toxicity LD_50_ (mg/kg)** | **Predicted toxicity class** | **Hepatotoxicity (Balance Accuracy 0.93)** | **Carcinogenicity (Balanced accuracy 0.81)** | **Immunotoxicity (Balanced accuracy 0.75)** | **Mutagenicity (Balanced accuracy 0.84)** | **Cytotoxicity (Balanced accuracy 0.85)** |
| --- | --- | --- | --- | --- | --- | --- | --- | --- | --- | --- | --- | --- | --- | --- | --- | --- | --- | --- | --- | --- | --- |
| **1.** | **Compound 2** | -2 | 15.199 | 0 | -2.618 | -5.026 | -9.029 | 0.135 | 0.033 | -7.285 | 1 | 0 | 3 | 2 | NA | NA | 0.95 | 0.74 | 0.99 | 0.98 | 0.53 |
| **2.** | **Compound 3** | -2 | 14.35 | 0 | -1.398 | -5.026 | -7.85 | 0.264 | 0.067 | -7.285 | 1 | 0 | 3 | 2 | 55 | 3 | 0.93 | 0.75 | 0.99 | 0.99 | 0.99 |
| **3.** | **Compound 4** | -2 | 23.697 | 0 | -2.769 | -5.001 | -7.06 | 0.803 | 0.223 | -6.603 | 1 | 0 | 3 | 2 | 55 | 3 | 0.95 | 0.74 | 0.99 | 0.98 | 0.71 |
| **4.** | **Compound 5** | -2 | 29.905 | 0 | -2.661 | -5.176 | -6.848 | 0.676 | 0.185 | -6.823 | 1 | 0 | 3 | 2 | NA | NA | 0.93 | 0.75 | 0.99 | 0.99 | 0.97 |
| **5.** | **Compound 6 (IMPHY001142)** | -2 | 20.466 | 0 | -2.12 | -4.982 | -9.453 | 0.064 | 0.015 | -8.36 | 1 | 0 | 3 | 2 | 55 | 3 | 0.95 | 0.74 | 0.99 | 0.98 | 0.71 |
| **6.** | **Compound 7 (10724418)** | -2 | 22.866 | 0 | -4.383 | -5.463 | -6.294 | 0.886 | 0.248 | -7.099 | 1 | 0 | 3 | 2 | 2000 | 4 | 0.95 | 0.74 | 0.99 | 0.98 | 0.71 |
| **7.** | **Compound 8 (SANC00684)** | -2 | 28.53 | 0 | -4.257 | -4.841 | -3.894 | 5.724 | 1.865 | -6.56 | 1 | 0 | 3 | 2 | NA | NA | 0.93 | 0.63 | 0.99 | 0.96 | 0.55 |
| **8.** | **Compound 10 (NPC159005)** | -2 | 12.954 | 0 | -1.45 | -4.843 | -10.05 | 0.014 | 0.003 | -9.472 | 1 | 0 | 3 | 2 | 8000 | 6 | 0.94 | 0.74 | 0.99 | 0.91 | 0.88 |
| **9.** | **Acarbose-derived Penta saccharide** | -2 | 8.378 | 0 | 1.982 | 1.827 | -6.944 | 0.008 | 0.002 | -11.544 | 1 | 0 | 3 | 2 | 2000 | 4 | 0.65 | 0.84 | 0.99 | 0.76 | 0.70 |

Note: NA: Not applicable.

**Table S3.** Top ten drug-disease targets for Gene Ontology analysis of biological processes, molecular functions, and cellular components

| **ID** | **Description** | **Gene Ratio** | **P value** | **Gene ID** |
| --- | --- | --- | --- | --- |
| **Biological Processes** | | | | |
| GO:0043434 | response to peptide hormone | 7/12 | 2.85981E-09 | GCK/MTOR/PIK3CA/PIK3R1/PIK3R2/PTPN1/NFKB1 |
| GO:0019216 | regulation of lipid metabolic process | 6/12 | 1.1294E-07 | ACACA/ACACB/MTOR/PIK3R1/PIK3R2/NFKB1 |
| GO:0030258 | lipid modification | 6/12 | 7.14487E-09 | ACACB/MTOR/PIK3CA/PIK3CB/PIK3R1/PIK3R2 |
| GO:0032868 | response to insulin | 6/12 | 9.25749E-09 | GCK/MTOR/PIK3CA/PIK3R1/PIK3R2/PTPN1 |
| GO:0071375 | cellular response to peptide hormone stimulus | 6/12 | 2.31495E-08 | GCK/PIK3CA/PIK3R1/PIK3R2/PTPN1/NFKB1 |
| GO:1901653 | cellular response to peptide | 6/12 | 7.04678E-08 | GCK/PIK3CA/PIK3R1/PIK3R2/PTPN1/NFKB1 |
| GO:0062012 | regulation of small-molecule metabolic process | 5/12 | 5.55566E-06 | ACACA/ACACB/GCK/MTOR/NFKB1 |
| GO:0032869 | cellular response to insulin stimulus | 5/12 | 1.7456E-07 | GCK/PIK3CA/PIK3R1/PIK3R2/PTPN1 |
| GO:0051897 | positive regulation of protein kinase B signaling | 5/12 | 5.30561E-08 | MTOR/PIK3CA/PIK3CB/PIK3R1/PIK3R2 |
| GO:0051896 | regulation of protein kinase B signaling | 5/12 | 3.0579E-07 | MTOR/PIK3CA/PIK3CB/PIK3R1/PIK3R2 |
| **Molecular Function** | | | | |
| GO:0030971 | receptor tyrosine kinase binding | 3/12 | 1.24194E-05 | PIK3R1/PIK3R2/PTPN1 |
| GO:0051219 | phosphoprotein binding | 3/12 | 2.04695E-05 | MTOR/PIK3R1/PIK3R2 |
| GO:1990782 | protein tyrosine kinase binding | 3/12 | 3.13703E-05 | PIK3R1/PIK3R2/PTPN1 |
| GO:0019903 | protein phosphatase binding | 3/12 | 0.000109357 | PIK3R1/PIK3R2/PTPN1 |
| GO:0019902 | phosphatase binding | 3/12 | 0.000238522 | PIK3R1/PIK3R2/PTPN1 |
| GO:0019207 | Kinase Regulator Activity | 3/12 | 0.000327074 | PIK3CA/PIK3R1/PIK3R2 |
| GO:0033218 | amide binding | 3/12 | 0.001698939 | ACACB/PIK3R1/CACNA1B |
| GO:0004674 | protein serine/threonine kinase activity | 3/12 | 0.00248087 | AKT3/MTOR/PIK3CA |
| GO:0016303 | 1-phosphatidylinositol-3-kinase activity | 2/12 | 1.75866E-05 | PIK3CA/PIK3CB |
| GO:0043560 | Insulin receptor substrate binding | 2/12 | 1.75866E-05 | PIK3CA/PIK3R1 |
| **Cellular Components** | | | | |
| GO:0005942 | phosphatidylinositol 3-kinase complex | 4/12 | 1.91289E-09 | PIK3CA/PIK3CB/PIK3R1/PIK3R2 |
| GO:0061695 | transferase complex, transferring phosphorus-containing groups | 4/12 | 1.24727E-05 | PIK3CA/PIK3CB/PIK3R1/PIK3R2 |
| GO:0019898 | extrinsic component of membrane | 4/12 | 2.63372E-05 | PIK3CA/PIK3CB/PIK3R1/PIK3R2 |
| GO:0005741 | mitochondrial outer membrane | 2/12 | 0.00593044 | ACACB/MTOR |
| GO:0031968 | organelle outer membrane | 2/12 | 0.00758268 | ACACB/MTOR |
| GO:0019867 | outer membrane | 2/12 | 0.007717515 | ACACB/MTOR |
| GO:0031932 | TORC2 complex | 1/12 | 0.007339604 | MTOR |
| GO:0038201 | TOR complex | 1/12 | 0.009166773 | MTOR |
| GO:0098554 | cytoplasmic side of endoplasmic reticulum membrane | 1/12 | 0.009166773 | PTPN1 |
| GO:0099524 | postsynaptic cytosol | 1/12 | 0.010990858 | MTOR |

**Table S4.** Analysis of the KEGG pathway

| **ID** | **Description** | **Gene Ratio** | **P value** | **Gene ID** |
| --- | --- | --- | --- | --- |
| hsa04910 | Insulin signalling pathway | 10/12 | 7.59381E-17 | AKT3/ACACA/ACACB/GCK/MTOR/PIK3CA/PIK3CB/PIK3R1/PIK3R2/PTPN1 |
| hsa04931 | Insulin resistance | 9/12 | 1.76578E-15 | AKT3/ACACB/MTOR/PIK3CA/PIK3CB/PIK3R1/PIK3R2/PTPN1/NFKB1 |
| hsa04152 | AMPK signaling pathway | 8/12 | 8.20568E-13 | AKT3/ACACA/ACACB/MTOR/PIK3CA/PIK3CB/PIK3R1/PIK3R2 |
| hsa05207 | Chemical carcinogenesis - receptor activation | 8/12 | 7.7574E-11 | AKT3/MTOR/PIK3CA/PIK3CB/PIK3R1/PIK3R2/CACNA1B/NFKB1 |
| hsa04930 | Type II diabetes mellitus | 7/12 | 8.25218E-14 | GCK/MTOR/PIK3CA/PIK3CB/PIK3R1/PIK3R2/CACNA1B |
| hsa05221 | Acute myeloid leukemia | 7/12 | 1.32579E-12 | AKT3/MTOR/PIK3CA/PIK3CB/PIK3R1/PIK3R2/NFKB1 |
| hsa04917 | Prolactin signaling pathway | 7/12 | 1.82462E-12 | AKT3/GCK/PIK3CA/PIK3CB/PIK3R1/PIK3R2/NFKB1 |
| hsa05230 | Central carbon metabolism in cancer | 7/12 | 1.82462E-12 | AKT3/GCK/MTOR/PIK3CA/PIK3CB/PIK3R1/PIK3R2 |
| hsa05212 | Pancreatic cancer | 7/12 | 3.31685E-12 | AKT3/MTOR/PIK3CA/PIK3CB/PIK3R1/PIK3R2/NFKB1 |
| hsa04211 | Longevity regulating pathway | 7/12 | 1.03811E-11 | AKT3/MTOR/PIK3CA/PIK3CB/PIK3R1/PIK3R2/NFKB1 |

**Table S5.** Crossdocking trials employed lignads from other PDB IDs.

| **PDB IDs** | **Compounds** | **Docking Score (Kcal/mol^-1^)** | **MMGBSA (Kcal/mol^-1^)** | **RMSD (Å)** |
| --- | --- | --- | --- | --- |
| **3BAJ** | **Balanitesin (Compound 1)** | **-14.406** | **-125.47** | **1.59** |
|  | **Compound 9 (SN0224203)** | **-13.019** | **-128.41** |  |
|  | **Acarbose-derived penta saccharide** | **-12.500** | **-81.275** |  |
| 1XD0 | Balanitesin (Compound 1) | -13.177 | -117.804 | 2.94 |
|  | Compound 9 (SN0224203) | -14.225 | -100.844 |  |
|  | Acarbose-derived penta saccharide | -12.721 | -85.062 |  |
| 2QV4 | Balanitesin (Compound 1) | -10.622 | -42.010 | 1.87 |
|  | Compound 9 (SN0224203) | -15.026 | -117.262 |  |
|  | Acarbose-derived penta saccharide | -13.020 | -72.251 |  |

**Table S6.** Known non-proteinic α-amylase inhibitors with docking score, binding free energies (MMGBSA) and their IC_50_ values of the compounds.

| **S.No.** | **Compound Name** | **IC_50 (_µM)** | **Docking Score (Kcal/mol)** | **MMGBSA (Kcal/mol)** | **Reference** |
| --- | --- | --- | --- | --- | --- |
|  | Carnosol | 59.92 | -4.472 | -42.585 | ^[39]^ |
|  | Rosmanol | 118.06 | -5.382 | -54.213 |  |
|  | (-)-3-O-galloylepicatechin | 739 | -8.087 | -57.834 | ^[40]^ |
|  | (-)-3-O-galloylcatechin | 401 | -7.445 | -54.055 |  |
|  | Ferulic acid | 9500 | -4.82 | -43.302 |  |
|  | bicyclo[2.2.0]hexane-2,3,5-triol | 471.78 | -4.28 | -34.116 | ^[41]^ |
|  | 3β-O-acetyl betulinic acid | 197.48 | -4.468 | -57.847 |  |
|  | 2,7-dihydroxy-4H-1-benzopyran-4-one | 525.99 | -4.364 | -28.751 |  |
|  | Luteolin | 360 | -6.153 | -46.763 | ^[42,43]^ |
|  | Fisetin | 19.6 | -6.509 | -56.177 |  |
|  | Quercetin | 500 | -7.005 | -56.426 |  |
|  | Myricetin | 380 | -6.346 | -38.72 |  |
|  | Eupafolin | 48.0 | -7.743 | -56.863 |  |
|  | 4’-Amino-4-hydroxychalcone | 26.89 | -3.102 | -34.642 |  |
|  | Cyanidin | 380 | -5.382 | -54.213 | ^[44]^ |
|  | Cyanidin-3-glucoside | 300 | -9.177 | -74.594 |  |
|  | Cyanidin-3-galactoside | >1000 | -7.495 | -64.81 |  |
|  | Cyanidin-3,5-diglucoside | >1000 | -12.935 | -102.945 |  |
|  | Lupeol | 46.81 | -4.016 | -54.198 | ^[17]^ |
|  | β-amyrin | 32.33 | -3.293 | -49.228 |  |
|  | α-amyrin | 66.07 | -4.545 | -68.251 |  |
|  | Betulin | 284.35 | -3.94 | -56.658 |  |
|  | Betulinic acid | 123.12 | -4.468 | -50.528 |  |
|  | Scopoletin | 82.53 | -4.615 | -40.389 |  |
|  | Chrysophanol | >400 | -5.465 | -32.664 | ^[45]^ |
|  | Physcion | >400 | -5.668 | -36.654 |  |
|  | Emodin | 184.7 | -5.615 | -39.498 |  |
|  | Daucosterol | 46.4 | -4.737 | -95.183 |  |
|  | Rhododendrin | 107.5 | -6.316 | -48.337 |  |
|  | Genistein | 1.39 | -5.688 | -49.691 |  |
|  | Pelargonidin | 2.067 | -5.876 | -61.605 |  |
|  | b-sitosterol | 372.31 | -5.245 | -74.683 | ^[46]^ |
|  | halfordin | 197.53 | -3.394 | -46.988 |  |
|  | methyl p-coumarate | 1238.90 | -3.351 | -25.554 |  |
|  | protocatechuic acid | 579.94 | -4.777 | -12.637 |  |
|  | Eriodictyol | 318.2 | -6.326 | -44.223 | ^[47]^ |
|  | Acacetin 7-O-b-D-glucoside | 337.1 | -4.914 | -66.063 |  |
|  | Buddleoside | 112.5 | -8.055 | -75.916 |  |
|  | Luteolin 7-O-glucuronide | 61.5 | -8.073 | -66.689 | ^[48]^ |
|  | luteolin 7-O-glucoside | 81.7 | -7.787 | -76.885 |  |
|  | diosmetin7-O-glucuronide | 76.3 | -7.745 | -72.785 |  |
|  | Rhamnetin | 73.9 | -7.078 | -58.873 | ^[49]^ |
|  | Hesperidin | 26.04 | -9.632 | -73.447 | ^[50]^ |
|  | Narirutin | 70.80 | -10.01 | -92.438 |  |
|  | Poncirin | 39.19 | -10.123 | -60.739 |  |
|  | Didymin | 31.62 | -4.067 | -98.861 |  |
|  | Naringin | 36.35 | -10.329 | -59.62 |  |
|  | Neoeriocitrin | 4.69 | -10.97 | -64.942 |  |
|  | 8-Geranylnaringenin | 15.38 | -5.301 | -55.024 | ^[51]^ |
|  | Baicalin | 1475.64 | -7.369 | -59.524 | ^[52]^ |
|  | 1,2,3-tri-O-galloyl-β-D-glucopyranose | 138.87 | -9.284 | -85.024 | ^[53]^ |
|  | 3,4,6-tri- O-galloyl-D-glucopyranose | 143.97 | -8.714 | -74.713 | ^[54]^ |
|  | (+)-catechin 7-O-β-D-glucopyranoside | 1635.72 | -8.577 | -55.175 | ^[55]^ |
|  | (+)-epicatechin 7-O-β-D-glucopyranoside | 857.632 | -8.577 | -55.175 |  |
|  | Penta-O-galloyl β-D-glucopyranose | 6.32 | -14.578 | -71.235 | ^[56]^ |
|  | 3-O-methyl quercetin | 152.09 | -6.098 | -44.869 | ^[57]^ |
|  | naringenin 7-O-β-D-glucopyranoside | 115.56 | -8.099 | -65.313 |  |
|  | Ursolic acid | 15.10 | -3.912 | -55.622 | ^[58]^ |
|  | Oleanolic acid | 125.60 | -3.865 | -50.631 |  |

**Table S7.** Root Mean Square Deviation (RMSD) and Root Mean Square Fluctuation (RMSF) statistics for the 3BAJ–compound 1, 3BAJ–SN0224203, and 3BAJ–standard complexes during molecular dynamics simulation.

|  | **3BAJ-compound 1 complex** | **3BAJ-SN0224203 complex** | **3BAJ-standard Complex** |
| --- | --- | --- | --- |
| **Root Mean Square Deviation** | | | |
| **Minimum** | 0.95 | 0.97 | 0.89 |
| **Maximum** | 2.20 | 2.21 | 2.49 |
| **Average** | 1.89 | 1.64 | 1.95 |
| **± SD** | 0.20 | 0.18 | 0.31 |
| **Root Mean Square Fluctuation** | | | |
| **Minimum** | 0.35 | 0.36 | 0.33 |
| **Maximum** | 3.42 | 5.78 | 4.22 |
| **Average** | 0.83 | 0.73 | 0.85 |
| **± SD** | 0.50 | 0.53 | 0.63 |
